# Functional Differentiation of Duplicated Flavonoid 3-*O*-Glycosyltransferases in the Flavonol and Anthocyanin Biosynthesis of *Freesia hybrida*

**DOI:** 10.3389/fpls.2019.01330

**Published:** 2019-10-18

**Authors:** Xiangyu Meng, Yueqing Li, Tongtong Zhou, Wei Sun, Xiaotong Shan, Xiang Gao, Li Wang

**Affiliations:** ^1^Key Laboratory of Molecular Epigenetics of MOE and Institute of Genetics & Cytology, Northeast Normal University, Changchun, China; ^2^Key Laboratory of Plant Physiology and Development Regulation, School of Life Science, Guizhou Normal University, Guiyang, China; ^3^National Demonstration Center for Experimental Biology Education, Northeast Normal University, Changchun, China

**Keywords:** glycosyltransferase, flavonoid, FhMYBF1, FhPAP1, divergence

## Abstract

Flavonols and anthocyanins are two widely distributed groups of flavonoids that occurred apart during plant evolution and biosynthesized by shared specific enzymes involved in flavonoid metabolism. UDP-glucose, flavonoid 3-*O*-glycosyltransferase (UF3GT), is one of the common enzymes which could catalyze the glycosylation of both flavonol and anthocyanidin aglycons simultaneously *in vitro*. However, whether and how *UF3GT* paralogous genes function diversely at the biochemical and transcriptional levels are largely unknown. Recently, *Fh3GT1* was identified to be a member of *UF3GT*s in *Freesia hybrida*. However, its expression patterns and enzymatic characteristics could not coincide well with flavonol accumulation. In an attempt to characterize other flavonoids, especially flavonol glycosyltransferase genes in *Freesia*, three closest candidate *UFGT* genes—*Fh3GT2*, *Fh3GT3*, and *Fh3GT4*—were mined from the *Freesia* transcriptomic database and isolated from the flowers of the widely distributed *Freesia* cultivar, Red River^®^. Based on bioinformatic analysis and enzymatic assays, *Fh3GT2* turned out to be another *bona fide* glycosyltransferase gene. Biochemical analysis further proved that Fh3GT2 preferentially glucosylated kaempferol while *Fh3GT1* controlled the glucosylation of quercetin and anthocyanidins. In addition, transfection assays demonstrated that *Fh3GT2* could be mainly activated by the flavonol regulator FhMYBF1 or the anthocyanin regulator FhPAP1, whereas *Fh3GT1* could only be activated by FhPAP1. These findings suggested that *Fh3GT*s might have functionally diverged in flavonoid biosynthesis at both the biochemical and transcriptional levels.

## Introduction

The derivation of the land-dwelling plant life exemplifies a fundamental transition in plant evolution. The early plants encountered diverse abiotic pressures from temperature, moisture, and UV radiation (Agati et al., 2012; Mouradov and Spangenberg, 2014; Vries and Archibald, 2018). Like many other organisms, plants adopt varied protective measures against the inimical environment. For example, the emergence of the specialized metabolites, flavonoids, is among the most general responses to versatile habitats (Waters, 2003). Flavonoids with typical C6-C3-C6 skeletons are phenolic compounds that are widely distributed in plants (Marais et al., 2006). According to differences of the C3 unit, flavonoids can be further categorized into several subclasses including flavonols, proanthocyanidins, and anthocyanins.

Increasing studies suggested that different flavonoid subclasses evolved sequentially (Moore, 1972; Rodriguez, 1986; Swain, 1986). To mention, flavonols that are common to plants can be traced back in early *Bryophyte* (Musci). Proanthocyanidins did not appear before the first vascular plants (ferns), while anthocyanins were deemed to appear with the emergence of angiosperms, e.g., flowering plants. Therefore, it is reasonable to speculate that flavonols play indispensable roles such as a UV protectant during the emergence and adaptation of plants to dry land (Burchard et al., 2010; Chomicki et al., 2015). Besides, flavonols can also function as chemical molecules in plants–plants or plants–microorganisms communication, auxin transportation, pollen germination, and tolerance against abiotic stress (Agati et al., 2012; Buer et al., 2013; Tan et al., 2013; Weston and Ulrike, 2013; Li, P. et al., 2016; Ramos et al., 2016). Consequently, flavonols have become one of the most widely distributed flavonoids in terrestrial plants (Mol et al., 1998; Taylor and Grotewold, 2005; Ferreyra et al., 2012).

Flavonol biosynthesis shares a general pathway with anthocyanin and proanthocyanidin synthesis. Generally, chalcone synthase (CHS) catalyzes the first step by transforming malonyl-CoA and 4-coumaroyl-CoA into chalcone. The subsequent isomerization of chalcone resulting in naringenin is promoted by chalcone isomerase (CHI). The naringenin is further hydroxylated by flavanone 3-hydroxylase (F3H) to generate dihydrokaempferol, which is subsequently catalyzed by flavonoid 3′-hydroxylase (F3′H) and flavonoid 3′,5′-hydroxylase (F3′5′H), yielding dihydroquercetin and dihydromyricetin, respectively. Dihydrokaempferol, dihydroquercetin, and dihydromyricetin are collectively referred to as dihydroflavonols, which can be further converted to different flavonol types by flavonol synthase (FLS). Alternatively, the dihydroflavonols can also be utilized by dihydroflavonol 4-reductase (DFR) to produce leucoanthocyanidins, which finally form anthocyanidins catalyzed by leucoanthocyanidin dioxygenase (LDOX). In addition, leucoanthocyanidins and anthocyanidins can also be converted to proanthocyanidins by leucoanthocyanidin reductase (LAR) and anthocyanidin reductase (ANR), respectively. Furthermore, flavonoid synthesized from the stated pathway undergoes further modifications, such as glucosylation, methylation, or acylation, in order to become more stable. In a given case, glycosylation is necessary for the flavonoids to enhance stability and solubility, as well as their subcellular localization (Vogt and Jones, 2000; Caputi et al., 2012; Sun et al., 2016). Generally, uridine diphosphate (UDP): flavonoid glycosyltransferases (UFGTs) are known as the obligatory enzymes that traffic the sugar molecules to the aglycones primarily (Dooner et al., 1991; Holton and Cornish, 1995; Martens and Mithöfer, 2005; Tanaka et al., 2010).

UDP-glucose: flavonoid 3-*O*-glycosyltransferases (UF3GTs), which utilize UDP-glucose as an activated donor of sugar moieties to the 3-position of flavonoid, are usually regarded as the first enzymes that catalyze the formation of glycosylated products. The UF3GTs belong to the superfamily characterized by a Plant Secondary Product Glycosyltransferase (PSPG box) composed of 44 conserved amino acids (Vogt and Jones, 2000; Caputi et al., 2012). Generally, the UF3GTs exhibit expansive substrate specificities by transferring UDP sugars to a series of flavonoids *in vitro* (Fukuchi-Mizutani et al., 2003; Sawada et al., 2005; Yonekura-Sakakibara et al., 2007). However, it is still an open question to interpreting the origin evolution of UF3GTs from different species or the functional divergence of different copies in the same plant considering the following two limitations. Firstly, genes encoding UF3GTs have been cloned and characterized from a variety of plants to date (Griesser et al., 2008; Kovinich et al., 2010; Ono et al., 2010; Montefiori et al., 2011; Song et al., 2015; Cui et al., 2016; Huang et al., 2018). However, less attention has been paid to non-dicotyledonous plants. Secondly, though the kinetic parameters of several UF3GTs have been investigated in detail, the enzyme specificities of UF3GTs from the same plant have yet to be largely formulated (Modolo et al., 2009; Kovinich et al., 2010; Hall et al., 2012). Again, studies on the function and evolution of UF3GTs in monocots are scarce, notwithstanding their possible agronomical importance.

The monocotyledonous *Freesia hybrida* in the Iridaceae family is originally native in South Africa and has been considered as one of the best popular cut flowers worldwide due to its sweet smell and versatile floral hues, such as purple, blue, red, yellow, white, and bicolor, amongst other floral traits. The rich flavonoid composed of flavonols, anthocyanins, and proanthocyanidins in flowers makes *Freesia* a prospective prototypical plant for investigating flavonoid biosynthesis and regulation in monocots. In our earlier studies, five anthocyanin aglycons—cyanidin, peonidin, delphinidin, petunidin, and malvinidin—as well as two flavonol types—quercetin and kaempferol derivatives—were detected in the floral organ of *F. hybrida* ‘Red River^®^’ (Sun et al., 2016). Till now, a versatile transient protoplast transfection assay was established based on the protoplasts isolated from *Freesia* callus (Shan et al., 2019a). Moreover, four regulatory genes and seven anthocyanin biosynthetic genes, including one glycosyltransferase gene *Fh3GT1* shown to prefer UDP-glucose as sugar donor to glycosylate anthocyanidins and flavonols, were cloned and characterized (Sui et al., 2011; Sun et al., 2015; Sun et al., 2016, Sun et al., 2017; Li, Y. et al., 2016; Li et al., 2017; Li et al., 2019; Ju et al., 2018; Shan et al., 2019b). However, the expression patterns and enzymatic characteristics of Fh3GT1 could not coincide well with flavonol accumulations during flower development, which suggested the existence of other potentially uncharacterized *UF3GT* genes preferentially linked to flavonol glycosylation.

To thoroughly characterize the flavonoid, especially flavonol-related glycosyltransferase genes in *Freesia*, another three *UFGT*s in addition to *Fh3GT1*, e.g., *Fh3GT2*, *Fh3GT3*, and *Fh3GT4*, were isolated from the flowers of the widely distributed *Freesia* cultivar, Red River^®^. The sequence alignment and phylogenetic analysis revealed that the *Fh3GT2*, *Fh3GT3*, and *Fh3GT4* genes were most likely grouped into the UF3GT family and encoded putative proteins that function as glycosyltransferases. However, the following enzymatic activity assays *in vitro* showed that only Fh3GT2 could catalyze the glycosylation of either anthocyanidins or flavonols. Compared to *Fh3GT1*, *Fh3GT2* had a declined expression pattern during flower development, which coincided with flavonol accumulation. Further kinetic analysis *in vitro* indicated that Fh3GT2 preferentially glycosylated kaempferol. This was also consolidated by comparing the correlations between the expression levels of Fh3GT2 and the respective flavonol and anthocyanin accumulations in Red River^®^ and Ambiance, respectively. Additionally, further transient protoplast transfection assays illustrated that Fh3GT2 had diverged with Fh3GT1 *in vivo*. *Fh3GT2* were markedly activated in *Freesia* protoplasts transiently overexpressing the flavonol-related regulator *FhMYBF1* or the anthocyanin-related regulator *FhPAP1*, whereas *Fh3GT1* was only upregulated in *FhPAP1* overexpressed protoplasts. Consistently, the promoter of *Fh3GT2* could be significantly activated by FhMYBF1 or FhPAP1, while *Fh3GT1* could only be regulated by FhPAP1. The outcome of this study not only plausibly expounded the *UF3GT*’s function in *Freesia* flavonoid biosynthesis but also shed light on the divergent evolution of *UF3GT*s in plants, especially in angiosperms, at both the biochemical and transcriptional levels.

## Materials and Methods

### Plant Materials and Growth Conditions

‘Red River^®^’ and ‘Ambiance’, cultivars of *F. hybrida* with red and white flowers, were grown in a greenhouse with a 14-h:10-h (light/dark) photoperiod at about 15°C. For detecting spatiotemporal expression profiles, five floral developmental stages and eight kinds of tissues or organs of Red River^®^ were sampled as mentioned in earlier studies (Sui et al., 2011; Sun et al., 2015; Sun et al., 2016; Sun et al., 2017; Li, Y. et al., 2016; Li et al., 2017; Gao et al., 2018; Ju et al., 2018; Li et al., 2019). For gene expression analysis between Red River^®^ and Ambiance, flowers fully bloomed on the first day were collected. All the materials were frozen in liquid nitrogen immediately and kept at −80°C for later use. For *Freesia* protoplast isolation, calluses were induced from the young inflorescence segments of Red River^®^ with a photoperiodism of 25°C with 14-h light and 10-h dark (Gao et al., 2010) and then subjected to protoplast isolation (Shan et al., 2019a). Rosette leaves of 4-week-old *Arabidopsis thaliana* (Columbia-0) grown under a 16-h light/8-h dark regime at 22°C were harvested for protoplast isolation.

### Nucleic Acid Extraction and cDNA Synthesis

NuClean Plant Genomic DNA Kit (CWBIO, Beijing, PRC) and OminiPlant RNA Kit (CWBIO, Beijing, PRC) were employed for DNA and RNA extraction following the manufacturer’s instruction accordingly. RNA or DNA contamination in DNA or RNA samples was eliminated by RNase or DNase I provided in the kit referring to the manufacturer’s protocol. Nanodrop 1000 spectrophotometry (Thermo Scientific, USA) was employed to detect the purity and concentration of the RNA samples. For cDNA synthesis, UEIris II RT-PCR System for First-Strand cDNA Synthesis Kit (US Everbright^®^ Inc., Suzhou, PRC) was used to reversely transcribe 500 ng of RNA into cDNA.

### Gene and Promoter Cloning

To mine the potential *Fh3GT*-like genes, the amino acid sequence of the earlier characterized Fh3GT1 (GenBank: ADK75021.1) was used as a bait sequence to conduct *in situ* TBLASTN screen against the *Freesia* transcriptomic database mentioned in our earlier studies (Li, Y. et al., 2016; Li et al., 2019). Manual BLASTX search of the National Center for Biotechnology Information (NCBI) was then carried out for the candidate gene screen. The specific primers (**Table S2**) were designed according to the predicted sequences and used to amplify the candidate genes, which were further sequenced after being ligated to pGEM-Teasy vector (Promega, Madison, WI). The amino acid sequences of GTs from *Freesia* and other species were processed by Clustal Omega algorithm for multiple sequence alignment (Sievers et al., 2011). For phylogenetic analysis, the GT proteins were handled by Clustal Omega with default parameters (http://www.ebi.ac.uk/Tools/msa/clustalo/) and further subjected to MEGA version 6 (Tamura, 2013) to generate the neighbor-joining tree with 1,000 bootstrap replications and handling gaps with pairwise deletion.

The genome sequences of *Fh3GT*s were amplified from *Freesia* genomic DNA using specific primers listed in **Table S2**. Moreover, the promoter of *Fh3GT2* was also cloned by using Genome Walking Kit (Takara, Dalian, PRC) following the method mentioned in our earlier studies (Li, Y. et al., 2016; Li et al., 2019). Finally, the gene structures were analyzed by comparing with their cDNA sequences. The 1,586 and 1,647 bp upstream of the ATG transcriptional start site of *Fh3GT1* and *Fh3GT2* were regarded as promoters, respectively. The tentative promoter sequences were subjected to PlantCARE analysis (Lescot et al., 2002).

### Heterologous Expression of Fh3GT Proteins in *E. Coli* and *In Vitro* Enzyme Assays

Heterologous expression of Fh3GT proteins in *Escherichia coli* was carried out as formerly narrated (Sun et al., 2015; Sun et al., 2016; Li et al., 2017; Gao et al., 2018). In brief, *Fh3GT* genes were subcloned into a *pET32a* vector and transformed into *E. coli* strain BL21 (DE3). The transformants were precultured at 37°C overnight. The precultured transformants were transferred to fresh media and cultured at 37°C for another 2.5 h. The recombinant proteins were then induced by 0.6 mM isopropyl-b-d-thiogalactopyranoside (IPTG) at 16°C for 18 h. Subsequently, the *E. coli* transformants were collected by centrifugation and then resuspended in phosphate-buffered saline (PBS, pH 7.4). The supernatant containing crude proteins was finally harvested by sonication before centrifugation. For protein purification, the PBS-equilibrated Ni Sepharose column (GE Healthcare) was employed to bond the N-terminally 6× His-tagged proteins. The purified proteins were then desalted in PBS and concentrated in Silica Gel Dryer (Sangon Biotech, Shanghai, PRC). The concentrated proteins were assessed by SDS-PAGE and Western blotting using ProteinFind Anti-His Mouse Monoclonal Antibody (TransGen Biotech, Beijing, PRC) and (horse radish peroxidase-conjugated Goat Anti-Mouse IgG (CoWin Biosciences, Beijing, PRC). The final concentrations of the purified proteins were detected by NanoDrop 1000 Spectrophotometer (ThermoFisher Scientific, USA).

To determine the substrate specialties of the Fh3GT proteins, standard recombinant enzyme assays were carried out in 200 µl of the reaction mixture consisting of 50 mM HEPES buffer (pH 8.0), 10 mM UDP-glucose, 100 µM flavonoid substrates (pelargonidin, petunidin, delphinidin, peonidin, cyanidin, malvidin, kaempferol, quercetin, and myricetin, Sigma), and 30 µg purified protein (Sun et al., 2016). The mixture was kept at 30°C for 5 min and subsequently mixed with 50 µl of 5% HCl solution. The mixture was centrifuged at 12,000 rpm for 5 min and the supernatant was infiltrated through a 0.22-µm membrane filter followed by analysis by HPLC system with an ACCHROM XUnion C18 column (250 mm × 4.6 mm, 5 µm). Solvent systems A (5% formic acid in H_2_O) and B (methanol) were used to elute the column at a flow rate of 1 ml min^−1^ with the following procedures: 0–10 min, 14–17% B; 10–35 min, 17–23% B; 35–60 min, 23–47% B; 60–67 min, 47–14% B; 67–70 min, 14% B. The products were detected at 520 nm for anthocyanins and 360 nm for flavonols.

For determination of *K*_m_ values, various concentrations of kaempferol, quercetin, and peonidin from 10 to 60 µM were employed as receptor substrates. Of UDP-glucose, 10 mM was employed as the donor substrate. The substrates were catalyzed with 4 µg of the purified enzymes in HEPES buffer for 2 min and analyzed by HPLC. All the other parameters were the same as the enzyme assays mentioned above.

### Analysis of Anthocyanins and Flavonols

The relative anthocyanin or flavonol contents in flowers of *F. hybrida* cultivars were analyzed according to the optimized approaches (Sun et al., 2016). Briefly, the flowers were ground to a powder in a mortar and 0.3-g samples were soaked in H_2_O/MeOH/HCl (75:24:1, *v*/*v*/*v*) at 4°C overnight in the dark. After centrifugation at 12,000 rpm for 10 min, the supernatant was infiltrated through a 0.22-µm membrane filter followed by analysis by HPLC system with an ACCHROM XUnion C18 column (250 × 4.6 mm, 5 µm). The HPLC parameters were the same as the enzyme assays aforementioned.

### Quantitative Real-Time PCR Analysis

To quantify the transcripts of *Fh3GT2* and *Fh3GT1*, specific primers were designed for quantitative real-time PCR (qRT-PCR) assays which were carried out in 10-μl reaction volumes with an ABI StepOne Plus Real-Time PCR System (USA). The total volume contained 5 μl SYBR Master Mix (TOYOBO, Japan), 0.5 μM of each primer, and 1 μl of cDNA templates. The cycling conditions were as follows: 95°C for 5 min, 45 cycles of 95°C for 10 s, 60°C for 10 s, and 72°C for 20 s. 18S rRNA was used as the internal reference and the gene expression levels were calculated with the formula 2^−ΔΔCт^ (Livak and Schmittgen, 2001).

### Transient Protoplast Assays

The GUS reporter vector *Fh3GT1-pro:GUS* and the modified human influenza hemagglutinin (HA)-tagged *pUC19* vector were depicted earlier (Li, Y. et al., 2016; Li et al., 2019). To construct the HA-tagged *FhPAP1* or *FhMYBF1* vector, the modified HA-tagged *pUC19* vector was digested by *Nde* I and *Afl* II and the backbone of *pUC19* was recovered and ready for further usage. The coding sequence of *FhPAP1* or *FhMYBF1* was amplified with specific primers (**Table S2**) and assembled with the recovered *pUC19* fragment by Minerva Super Fusion Cloning Kit (US Everbright^®^ Inc., Suzhou, PRC). For constructing *Fh3GT2-pro:GUS* vector, the earlier used *Fh3GT1-pro:GUS* was digested by *Pst* I and *Sac* I and the backbone of the vector was recovered. The promoter of *Fh3GT2* was amplified with specific primers (**Table S2**) and assembled by Minerva Super Fusion Cloning Kit following the manufacturer’s instruction.

The GoldHi EndoFree Plasmid Maxi Kit (CWBIO, Beijing, PRC) was employed to prepare the plasmids used in the protoplast transfection assays. The extracted plasmids were further concentrated by 2.84 ml of isopropanol and 0.84 ml of 5 M NaCl (Li, Y. et al., 2016; Li et al., 2019). Protoplast isolation, transfection, and GUS activity assays were executed by referring to well-established protocols (Wang and Chen, 2014; Zhou et al., 2014; Li, Y. et al., 2016; Shan et al., 2019a). Briefly, protoplasts were isolated from *Arabidopsis* rosette leaves or *Freesia* calluses. The protoplasts were transfected by different combinations of plasmids using PEG3350 (Solarbio Life Science, Beijing, PRC) and were incubated at 22°C for 20–22 h under darkness. The protoplasts were further used for quantifying gene transcripts by qRT-PCR or detecting the GUS activities with a Synergy™ HT microplate reader (BioTEK, www.biotek.com).

## Results

### Isolation and Characterization of *Fh3GT*-Like Genes From *F. hybrida*

**Figure 1:**
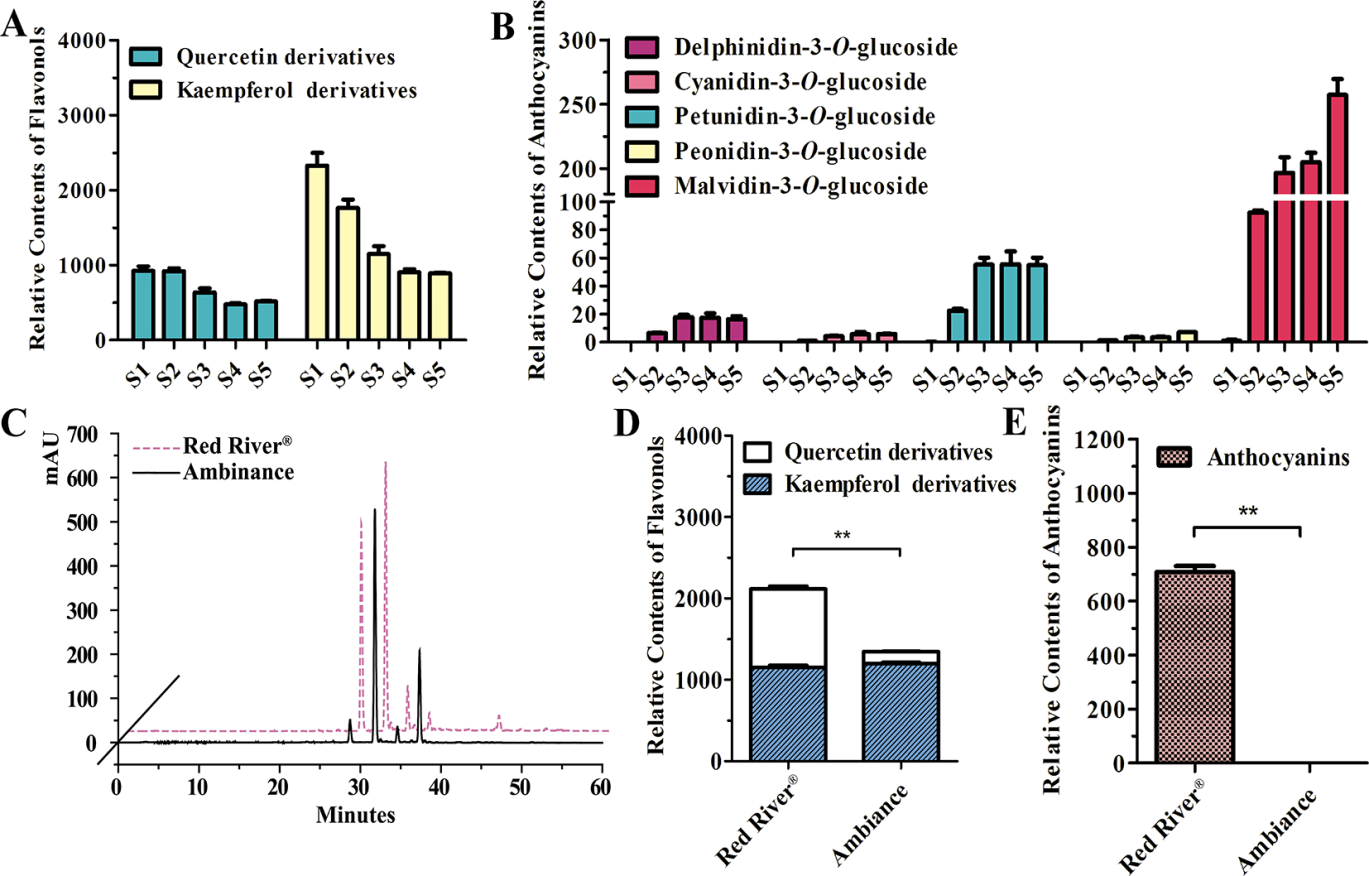
Quantitative analysis of anthocyanins and flavonols. **(A** and **B)** Relative contents of flavonols and anthocyanins during flower development of Red River^®^. **(C)** Peak patterns of flower flavonols analyzed by HPLC. **(D** and **E)** Relative contents of flavonols and anthocyanins in flowers of Red River^®^ and Ambiance. Relative content was represented by the peak area of HPLC analysis. *S1*–*S5* represented the different developmental stages of flowers. Data represented the means ± SD of three biological replicates. T test was carried out to analyze the significant difference (**P < 0.01).

As noted earlier, there were two kinds of flavonols and five kinds of anthocyanins that mounted up in the flowers of *Freesia* cultivar Red River^®^ (Li, Y. et al., 2016; Sun et al., 2016). The flavonols and anthocyanins were further extracted and analyzed by HPLC to ascertain accumulation profiles throughout the flowering stages. The results showed that the flavonol derivatives decreased as flower was developing, whereas anthocyanins gradually increased, conceivably implying a competitive relationship between flavonol and anthocyanin biosynthesis (**Figures 1A, B**). To further decipher the correlations between anthocyanin and flavonol biosynthesis, two common cultivars of *F. hybrida*, Red River^®^ and Ambiance, with red flowers and white flowers were used, respectively. Consequently, high-performance liquid chromatography–mass spectrometry (HPLC-MS) analysis showed that Red River^®^ and Ambiance flowers accumulated similar types of flavonol derivatives (**Figure 1C**). In the Ambiance petals, HPLC-MS analysis also identified two kinds of flavonol derivatives: quercetin and kaempferol (**Figure S1**). Outstandingly, in both Red River^®^ and Ambiance cultivars, relatively equal amounts of kaempferol derivatives were accumulated compared to contents of quercetin derivatives (**Figure 1D**). Paralleled with its colorless phenotype, Ambiance synthesized non-detectable anthocyanins in petals when matched with its Red River^®^ counterpart (**Figure 1E**). Moreover, the flavonol and anthocyanin accumulating patterns were subsequently analyzed in different tissues or organs of Red River^®^. The results showed that the anthocyanins were mainly accumulated in the reproductive tissues or organs, whereas the flavonols were detected in all the aerial tissues or organs of Red River^®^ (**Figure S2**).

**Figure 2:**
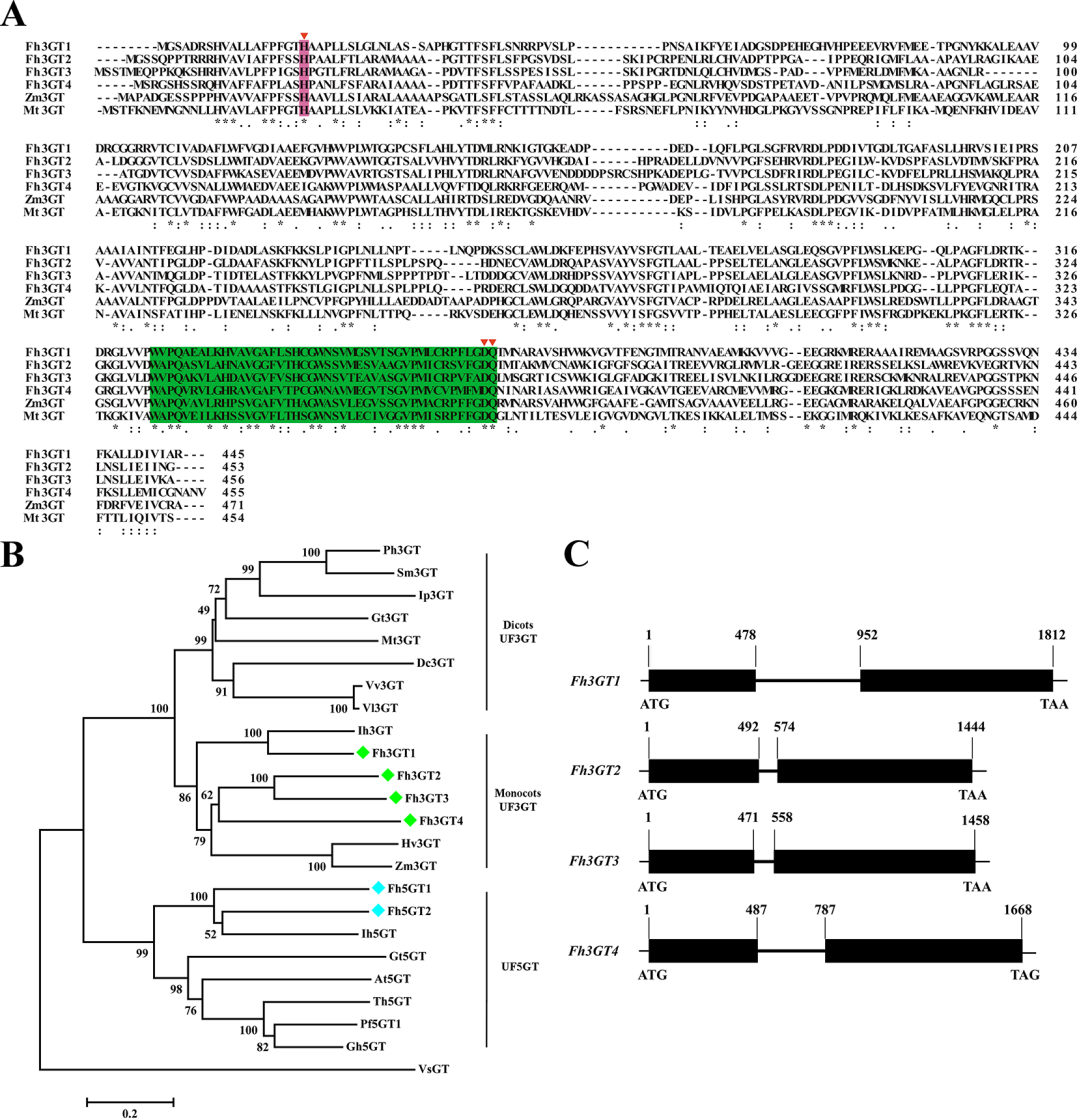
Molecular analysis of Fh3GT proteins. **(A)** Protein alignment of the potential Fh3GT proteins and their homologs in other species. *Numbers* indicated the position of the last amino acid in each line. The conserved His and PSPG box were *shaded in different colors*. *identical amino acids;: or., similar amino acids. **(B)** Phylogenetic analysis between the predicted Fh3GT proteins and other glycosyltransferases. *Numbers* indicated bootstrap values for 1,000 replicates. *Freesia* proteins were indicated as *green* and *blue diamonds*. The GenBank accession numbers of the protein sequences used were as follows: *Fressia hybrida* Fh3GT1 (ADK75021.1), Fh3GT2 (MK945761); *Medicago truncatula* Mt3GT (XP_003610163); *Vitis vinifera* Vv3GT (AF000371); *Gentiana triflora* Gt3GT (D85186), Gt5GT (BAG32255); *Petunia hybrida* Ph3GT (AB027454); *Ipomea purpurea* Ip3GT (AF028237); *Iris hollandica* Ih3GT (BAD83701), Ih5GT (BAD06874); *Zea mays* Zm3GT (CAA31856); *Hordeum vulgare* Hv3GT (AA33729); *Solanum melongena* Sm3GT (Q43641); *Dianthus caryophyllus* Dc3GT (BAD52003); *Vitis labrusca* Vl3GT (ABR24135); *Perilla frutescens* Pf5GT1 (BAA36421); *Torenia hybrid* Th5GT (BAC54093); *Arabidopsis thaliana* At5GT (NP_193146); *Glandularia hybrida* Gh5GT (BAA36423); *Viral steroid* VsGT (X99073). **(C)** Genome structures of the potential *Fh3GT*-like genes. *Blank boxes* indicated the extrons and *numbers* indicated the position of nucleotide in the coding sequence.

To isolate the potential glucosyltransferases participating in flavonoid especially flavonol biosynthesis, the amino acid sequence of Fh3GT1 hitherto described in our earlier studies was used as a bait probe to thoroughly search the would-be flavonoid 3-*O*-glucosyltransferases in *F. hybrida* (Sui et al., 2011; Sun et al., 2016; Sun et al., 2017). Afterwards, another 38 putative unigenes were mined from the *Freesia* floral transcriptomic database and predicted as glycosyltransferases. The 38 unigenes and Fh3GT1 were subjected to a preliminary phylogenetic analysis together with glycosyltransferases from other plants. Consequently, another three putative sequences encoding 3-*O*-glycosyltransferases were isolated and projected to be *Fh3GT*-like genes (tentatively named *Fh3GT2*, *Fh3GT3*, and *Fh3GT4*, respectively) in *F. hybrida* (**Figure S3**). Further analysis in detail indicated that their coding proteins shared identities with the formerly characterized Fh3GT1, implying possible comparable functions (**Table S1**). Sequence alignment with flavonoid 3-*O*-glucosyltransferase genes from *Zea mays* and *Medicago truncatula* showed the conserved His 20 and PSPG box in these proteins (**Figure 2A**), further reinforcing our notion that Fh3GT2, Fh3GT3, and Fh3GT4 might perform functions in flavonoid biosynthesis (Caputi et al., 2012).

To search the homologies of the three *Freesia* proteins to other known UFGT proteins, an alternative phylogenetic map was constructed (**Figure 2B**). The UF3GT and UF5GT proteins belonged to diverse subclades, implicating their different roles in eukaryotic organisms. Moreover, UF3GT proteins from monocots and eudicots fell into two branches, while the entire Fh3GT-like proteins clustered within the monocot clade. More perfectly, Fh3GT1 was most similar to *Iris* × *hollandica* Ih3GT, while Fh3GT2, Fh3GT3, and Fh3GT4 clustered together due to significant similarities. Moreover, their genome structures were examined in order to elucidate the differences between the *Fh3GT*-like genes. The results showed that they shared similar genome structures with only one intron in each coding sequence irrespective of the intron length (**Figure 2C**). Conclusively, the abovementioned results pointed out that the three newly cloned *Fh3GT*-like genes potentially played parts in flavonoid biosynthesis in *Freesia*.

### Fh3GT2 Turned Out to Be Another *Bona Fide* Glucosyltransferase in *F. hybrida*

**Figure 3:**
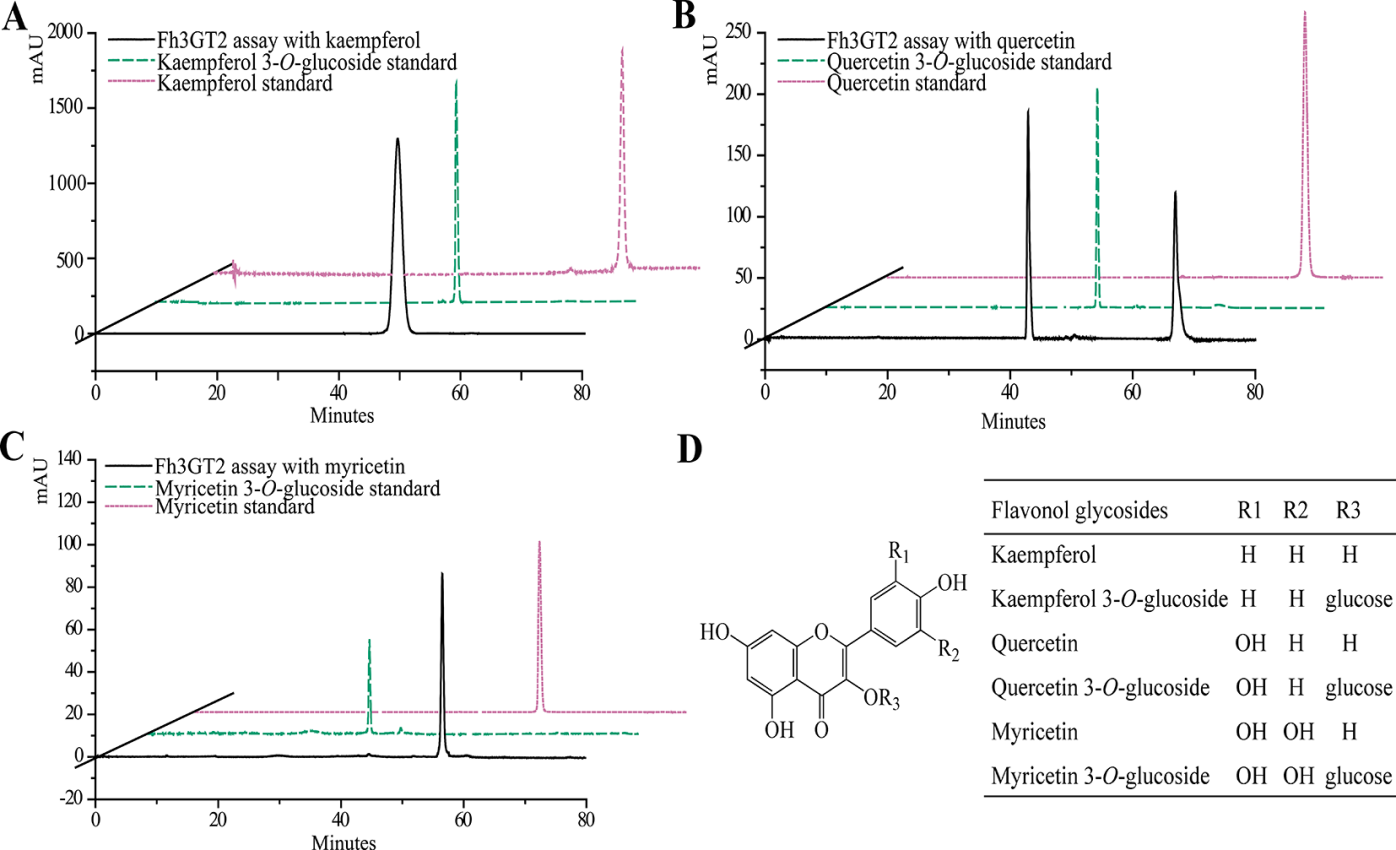
*In vitro* enzyme activity assays of Fh3GT2 towards flavonol aglycones. **(A**–**C)** *In vitro* enzyme activity assays of Fh3GT2 towards kaempferol, quercetin, and myricetin, respectively. **(D)** Molecular structures of flavonol derivatives. The recombinant Fh3GT2 extracted from *Escherichia coli* was reacted with UDP-glucose and flavonol aglycones. Identification of the products was confirmed based on the standard substance and the relative retention time.

**Figure 4:**
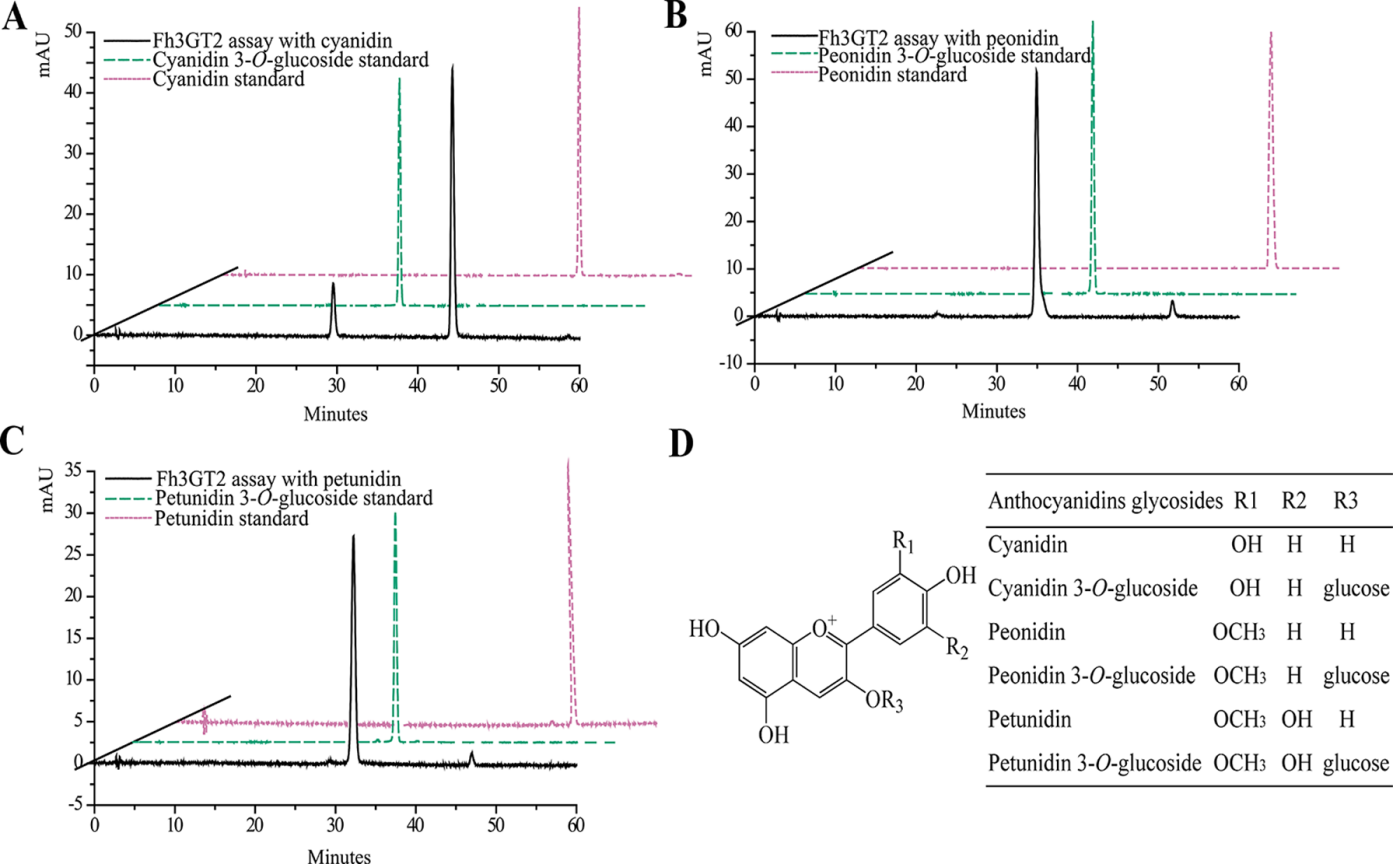
*In vitro* enzyme activity assays of Fh3GT2 towards anthocyanin aglycones. **(A**–**C)** *In vitro* enzyme activity assays of Fh3GT2 towards cyanidin, peonidin, and petunidin, respectively. **(D)** Molecular structures of anthocyanin derivatives. The recombinant Fh3GT2 extracted from *Escherichia coli* was reacted with UDP-glucose and anthocyanin aglycones. Identification of the products was confirmed based on the standard substance and the relative retention time.

Earlier researches show that Fh3GT1 mainly transfers UDP-glucose to all the tested anthocyanidin and flavonol aglycones in *Freesia* flowers (Sun et al., 2016). Moreover, the flavonol aglycone myricetin, which was not accumulated in *Freesia* flowers, could also be glucosylated by Fh3GT1 (**Figure S4**). Herein, the substrate specificity assays were conducted using UDP-glucose as the sugar donor. Similarly, in order to further elucidate the enzymatic properties of Fh3GT2, Fh3GT3, and Fh3GT4, recombinant proteins were extracted from *E. coli* and purified by the GE Healthcare Ni Sepharose column (**Figure S5**). The substrate specificities of the purified Fh3GT proteins in catalyzing UDP-glucose to substrates including three basic flavonols and six basic anthocyanidins were evaluated. As results, Fh3GT2 could efficiently glucosylate flavonol aglycones kaempferol and quercetin, except myricetin (**Figure 3**). Moreover, petunidin and peonidin could also be glucosylated by Fh3GT2, with high efficiencies, while cyanidin was partly glucosylated to cyanidin 3-*O*-glucoside (**Figure 4**). In contrast, pelargonidin, delphinidin, or malvidin might not be the natural substrate of Fh3GT2, as indicated (**Figure S6**). Unexpectedly, neither Fh3GT3 nor Fh3GT4 could transfer glucose to either flavonols or anthocyanidins aforementioned (**Figure S7**), denoting that they might be pseudofunctional in the glucosylation of flavonol or anthocyanidin.

### Expression Profiles of *Fh3GT2* and *Fh3GT1* Suggested Functional Differentiation in the Glucosylation of Flavonol and Anthocyanidin

**Figure 5:**
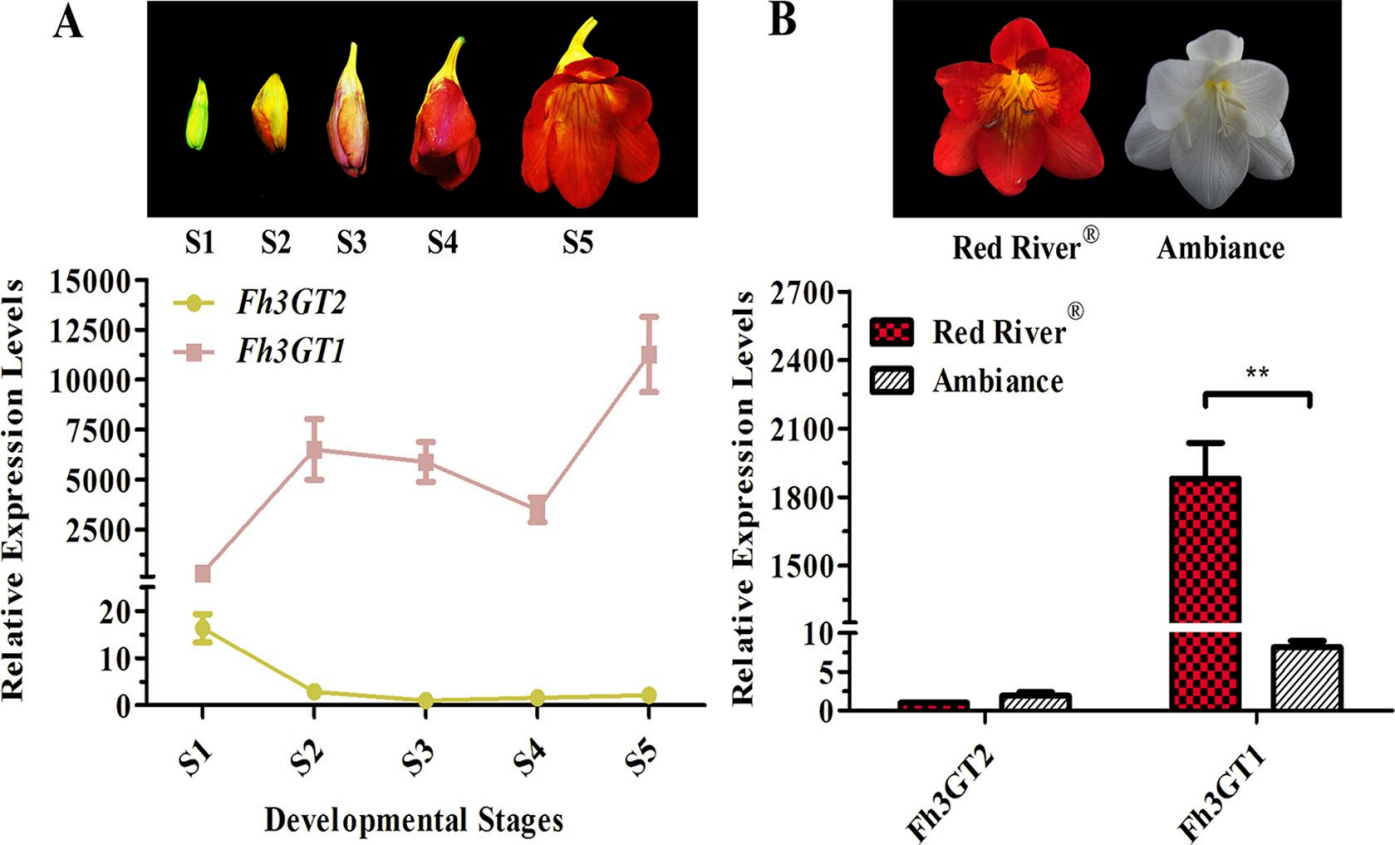
Expression profiles of *Fh3GT2* and *Fh3GT1* in different flower developmental stages and different *Freesia* cultivars. **(A)** Expression levels of *Fh3GT2* and *Fh3GT1* in flowers at different developmental stages. *S1*–*S5* represented the different developmental stages of flowers. **(B)** Expression profiles of *Fh3GT2* and *Fh3GT1* in *Freesia* cultivar Red River^®^ and Ambiance. Data represented the means ± SD of three biological replicates. *T* test was carried out to analyze the significant difference (**P < 0.01).

To additionally evaluate the correlations between the transcripts of *Fh3GT2* and flavonol or anthocyanin biosynthesis, quantitative real-time PCR was used to quantify the transcripts of *Fh3GT2* during floral developments and in the two *Freesia* cultivars. Moreover, *Fh3GT1* was also included comparably. *Fh3GT2* exhibited high expression levels in non-pigmented buds and decreased as flower was developing; this resembled the flavonol accumulation in *Freesia* plant (**Figures 1A**, **5A**). However, the opposite expression pattern was observed for *Fh3GT1*, which showed a generally upward trend consistent with the accumulation of anthocyanins (**Figures 1B**, **5A**; Sun et al., 2016). The results indicated that Fh3GT2 and Fh3GT1 might function differentially in flavonoid biosynthesis, although they could glucosylate a series of aglycones. Further expression analysis in the blooming flowers of both cultivars revealed a relatively stable *Fh3GT2* expression pattern corresponding to the identical kaempferol contents between the two cultivars. However, the transcripts of *Fh3GT1* were nearly 200-fold higher in Red River^®^ flowers than those in Ambiance flowers, which resembled well the different contents of anthocyanins and quercetin derivatives in these cultivars (**Figures 1D, E** and **5B**). The results justified the speculation that Fh3GT2 might mainly prefer kaempferol as a substrate, while Fh3GT1 probably glycosylated anthocyanidins and quercetin *in vivo*.

The spatial expression patterns of Fh3GT3 and Fh3GT4 were also assessed despite no catalytic activity of Fh3GT3 or Fh3GT4 being detected in glucosylating anthocyanidins and flavonols. Comparably, *Fh3GT3* and *Fh3GT4* had relatively higher expression levels than *Fh3GT2* in almost all the detected tissues or organs (**Figure S8**), implying undeciphered roles of Fh3GT3 and Fh3GT4.

### Fh3GT2 and Fh3GT1 *in Vitro* Bioassay Revealed Different Substrate Preferences

Previous substrate specificity assays discovered that Fh3GT1, a versatile glycosyltransferase, generally glucosylated delphinidin and quercetin most efficiently in sufficient substrates (**Table 1**; Sun et al., 2016). Moreover, the relative activities of Fh3GT2 towards several substrates were also assessed in the presence of UDP-glucose. As Fh3GT2 showed minor catalytic activities towards delphinidin, pelargonidin, and malvidin (**Figure S6**), the relative activity of Fh3GT2 in the respective substrate was not assessed. Evidently, Fh3GT2 could glucosylate quercetin most efficiently in sufficient substrates; nonetheless, kaempferol and peonidin exhibited relatively higher rates of glucosylation than petunidin and cyanidin (**Table 1**).

**Table 1:** Relative activities of Fh3GT2 and Fh3GT1 towards several substrates.

Substrate	Sugar Donor	Product	Relative Activity of Fh3GT2 (%)	Relative Activity of Fh3GT1 (%)
Quercetin	UDP-Glu	Quercetin 3-*O*-glucoside	100^a^	100^a^
Kaempferol	UDP-Glu	Kaempferol 3-*O*-glucoside	38.06	41.56
Peonidin	UDP-Glu	Peonidin 3-*O*-glucoside	36.68	31.95
Petunidin	UDP-Glu	Petunidin 3-*O*-glucoside	24.07	12.05
Cyanidin	UDP-Glu	Cyanidin 3-*O*-glucoside	2.18	27.94
Delphinidin	UDP-Glu	Delphinidin 3-*O*-glucoside	ND^b^	32.73
Pelargonidin	UDP-Glu	Pelargonidin 3-*O*-glucoside	ND	14.16
Malvidin	UDP-Glu	Malvidin 3-*O*-glucoside	ND	8.03

100 μM substrate and 10 mM sugar were used and the reaction was incubated at 30°C for 5 min.

Activity values are the mean of three independent determinations.

a Relative activity was calculated by the activity toward quercetin as 100%.

b ND indicated that we have not carried out the assays.

Though previous studies revealed that Fh3GT1 could glucosylate quercetin most efficiently when compared to delphinidin and peonidin, it was further designated to prefer catalyzing the glucosylation of anthocyanidins and quercetin *in vivo* considering its kinetic parameters and the cooperativities between substrate accumulation and gene expression, as well as the recovery assays in the *Arabidopsis 3gt* mutant (Sun et al., 2016). To further decipher the characteristics of Fh3GT2 and the discrepancies between Fh3GT2 and Fh3GT1, the kinetic parameters of recombinant Fh3GT2 as well as Fh3GT1 were investigated with the receptor of kaempferol, quercetin, and peonidin in the presence of UDP-glucose. As results, the recombinant Fh3GT2 exhibited the highest affinity (*K*_m_) for kaempferol (0.13 ± 0.01 μM) and a relatively high affinity for quercetin (16.62 ± 0.03 μM) when compared with that of peonidin (33.02 ± 2.70 μM; **Table 2**). As for Fh3GT1, it preferred kaempferol (45.38 ± 3.90 μM), peonidin (54.82 ± 2.81 μM), and quercetin (96.38 ± 5.03 μM), in that order. Moreover, kaempferol also displayed the highest conversion rate for both Fh3GT2 (*K*_cat_, 0.73 ± 0.0001 s^−1^) and Fh3GT1 (*K*_cat_, 0.0016 ± 0.0001 s^−1^), respectively. However, Fh3GT2 showed the highest catalytic efficiency (*K*_cat_/*K*_m_) in transferring UDP-glucose to the 3-position of kaempferol, which was 48.7-fold and 152.1-fold higher than those of quercetin and peonidin. Comparatively, Fh3GT1 had a relatively higher catalytic efficiency for quercetin, while the catalytic efficiency of Fh3GT2 towards kaempferol was 13,651.3-fold higher than that of Fh3GT1. The results further suggested that the recombinant Fh3GT2 preferred to transfer UDP-glucose to flavonol aglycones (mainly for kaempferol) rather than glucosylate anthocyanidins. Comparably, Fh3GT1 inclined to glucosylate quercetin and anthocyanidins according to its similar catalytic efficiency towards quercetin and peonidin (**Table 2**) and its extremely higher expression levels than *Fh3GT2*.

**Table 2:** Kinetic parameters of recombinant Fh3GT2 and Fh3GT1.

Substrate	*K*_m_ (μM)		*K*_cat_ (s^−1^)		*k*_cat_/*K*_m_ (s^−1^ M^−1^)	
	Fh3GT2	Fh3GT1	Fh3GT2	Fh3GT1	Fh3GT2	Fh3GT1
Kaempferol	0.13 ± 0.01	45.38 ± 3.90	0.73 ± 0.0001	0.0016 ± 0.0001	546.05 × 10^4^	0.04 × 10^4^
Quercetin	16.62 ± 0.03	96.38 ± 5.03	1.86 ± 0.0023	4.51 ± 0.0390	11.19 × 10^4^	4.69 × 10^4^
Peonidin	33.02 ± 2.70	54.82 ± 2.81	1.18 ± 0.0970	0.49 ± 0.0220	3.59 × 10^4^	0.91 × 10^4^

Data are the means ± standard deviation (SD) of three independent experiments.

### *Fh3GT2* and *Fh3GT1* Were Differentially Regulated by Either Flavonol-Specific or Anthocyanin-Specific Regulators in *F. hybrida*

**Figure 6:**
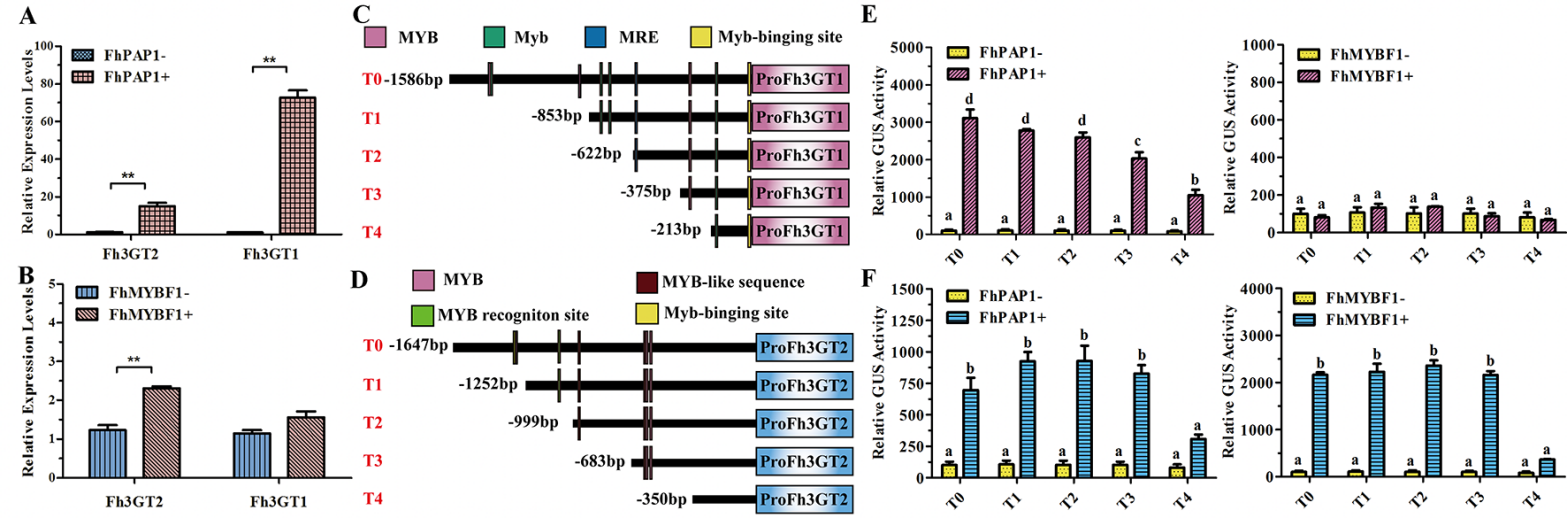
*Fh3GT2* and *Fh3GT1* were differentially regulated by FhPAP1 and FhMYBF1. **(A** and **B)** Relative expressions of *Fh3GT2* and *Fh3GT1* in *Freesia* protoplasts transiently expressing anthocyanin-specific *FhPAP1* or flavonol-specific *FhMYBF1*. **(C** and **D)** Schematic diagram of differently truncated Fh3GT promoters and the potential MYB binding sites in the promoter. The predicted MYB binding sites were presented as *colorful boxes*. **(E** and **F)** Promoter activations of *Fh3GT2* and *Fh3GT1* by FhPAP1 or FhMYBF1. The gene expression levels were quantified by qRT-PCR. The promoter activation abilities by the respective regulators were determined by the relative GUS activities. Data represented the mean ± SD of three replicates. *T* test was carried out to analyze the significant difference in **(A** and **B)** (**P < 0.01). One-way ANOVA was carried out to compare statistical differences in **(E** and **F)** (Ducan, *P* < 0.05).

In model plant *Arabidopsis*, the flavonol and anthocyanin biosynthetic pathways are regulated by different MYB regulators. Briefly, flavonols in *Arabidopsis* are activated by functionally redundant R2R3-MYB regulatory genes (*AtMYB11*, *AtMYB12*, and *AtMYB111*), whereas the accumulation of anthocyanins requires other sets of MYB regulators, i.e., AtPAP1, AtPAP2, AtMYB113, and AtMYB114 (Jaakola, 2013; Xu et al., 2015). In *Freesia*, two MYB regulators designated FhPAP1 and FhMYBF1 were characterized to regulate anthocyanin and flavonol biosynthesis, respectively (data unpublished). To further identify the divergent roles between Fh3GT2 and Fh3GT1, *Freesia* protoplasts transiently overexpressing *FhPAP1* or *FhMYBF1* were gathered and detected by qRT-PCR to investigate whether the two glucosyltransferase genes could be differentially regulated by these two regulators. As the results showed in **Figure 6A**, FhPAP1 could significantly activate either *Fh3GT2* or *Fh3GT1*. In contrast, FhMYBF1 could only activate the expression of *Fh3GT2* (**Figure 6B**). To further consolidate the results, the promoters of *Fh3GT2* and *Fh3GT1* were cloned and analyzed by PlantCARE (**Figures 6C, D**). The promoters were truncated orderly, referring to the possible MYB binding sites, and constructed as reporter constructs to promote *GUS* expressions. Subsequently, transiently expressing *FhPAP1* or *FhMYBF1* in combination with serials of *GUS* reporter constructs promoted by *FreesiaFh3GT2* or *Fh3GT1* promoters would decipher the possible binding positions of FhPAP1 and FhMYBF1. As the results showed in **Figures 6E, F**, both FhPAP1 and FhMYBF1 mostly recognized the *cis*-elements between T3 and T4 of *Fh3GT2* or *Fh3GT1*. The same results were also concluded that FhMYBF1 could only activate *Fh3GT2*, while FhPAP1 could significantly regulate *Fh3GT1* or *Fh3GT2*. However, *Fh3GT1* might be the primary preference of FhPAP1 as the induction was significantly higher than that of *Fh3GT2*. Consistent with the gene expression and biochemical analysis aforementioned, the results here further validated that Fh3GT2 mainly took part in flavonol biosynthesis, while Fh3GT1 preferred anthocyanin biosynthesis.

## Discussion

Glycosylation is usually the last step in the flavonoid biosynthetic pathway. The UF3GT that transfers UDP-activated sugars to flavonoid compounds is considered as a key enzyme for flavonoid modification *in vivo* to confer stability (Vogt and Jones, 2000; Zhang et al., 2014). In our previous studies, Fh3GT1 was proven to glycosylate a repertoire of flavonoid substrates both *in vitro* and in *Arabidopsis*.

In our determination to understand the *Freesia* flavonoid glycosylation system, the *Freesia* flower transcriptomic database was screened focusing on genes that were annotated as UDP-glucose: flavonoid 3-*O*-glucosyltransferase. However, the precise lineage evolution of UFGTs makes activity prediction based on sequence match alone a difficult undertaking. In the case of *Freesia*, prediction is even tougher due to a few isolated and functionally characterized flavonoid glycosyltransferases in monocot species. Nevertheless, another three putative paralogous sequences of *Fh3GT1* were cloned and seemed to be UF3GT-like proteins from *Freesia*, though their encoded proteins exhibited low sequence identity with Fh3GT1 (**Table S1**). In contrast, high protein sequence identities were observed between UFGTs from the same species in other plants. For example, AtUGT78D2 and AtUGT78D3 from *A. thaliana* showed 75% amino acid sequence identity (Kim et al., 2013). Moreover, the eight flavonoid-related VvGTs isolated from *Vitis vinifera* had appreciable sequence identities (>51.3%) between each other (Ono et al., 2010). Herein, the relatively low sequence similarities between Fh3GT1 and other Fh3GTs might imply the functional differentiations among these proteins. Multiple sequence alignments indicated that all the three proteins as well as the formerly characterized Fh3GT1 had typical domains of glycosyltransferases including the PSPG box responsible for the recognition of the sugar donor (**Figure 2**) (Ross et al., 2001; Gachon et al., 2005; Caputi et al., 2012). To further hypothesize the functions of Fh3GTs in *Freesia*, a phylogenetic tree was assembled using UFGT sequences obtained from other plants. Fh3GT2, Fh3GT3, and Fh3GT4 phylogenetically placed in a branch containing UF3GTs from other monocots, doubtless suggesting that they share 3-*O*-glycosyltransferase-like catalytic properties. Numerous studies revealed that a single glycosyltransferase could glycosylate manifold substrates, and a substrate could be glycosylated by multiple enzymes (Bönisch et al., 2014a; Bönisch et al., 2014b). Consistent with our studies, Fh3GT1 was shown to glycosylate quercetin, kaempferol, malvidin, petunidin, peonidin, pelargonidin, cyanidin, and delphinidin with UDP-glucose moiety or transfer UDP-galactose to cyanidin and delphinidin (Sun et al., 2016). Similarly, Fh3GT2 catalyzed the glucosylation of several aglycones such as kaempferol, quercetin, cyanidin, petunidin, peonidin, and malvidin, which validated the assumption that Fh3GT2 functioned like an alternative UF3GT with UDP-glucose as the sugar contributor in *F. hybrida*. Moreover, Fh3GT2 could not use myricetin, but could accept quercetin and kaempferol as substrates. Also, myricetin derivatives were not detected when we checked the flavonoid contents. However, the substrate specificities of GTs might not be the primary cause of flavonoid contents as Fh3GT1, which could glucosylate myricetin, was highly expressed in flowers (**Figures 5**, **S4**). Actually, our preliminary studies have indicated that the substrate competition between FLS and DFR might be the cause of the myricetin derivative deficiency (unpublished data).

**Figure 7:**
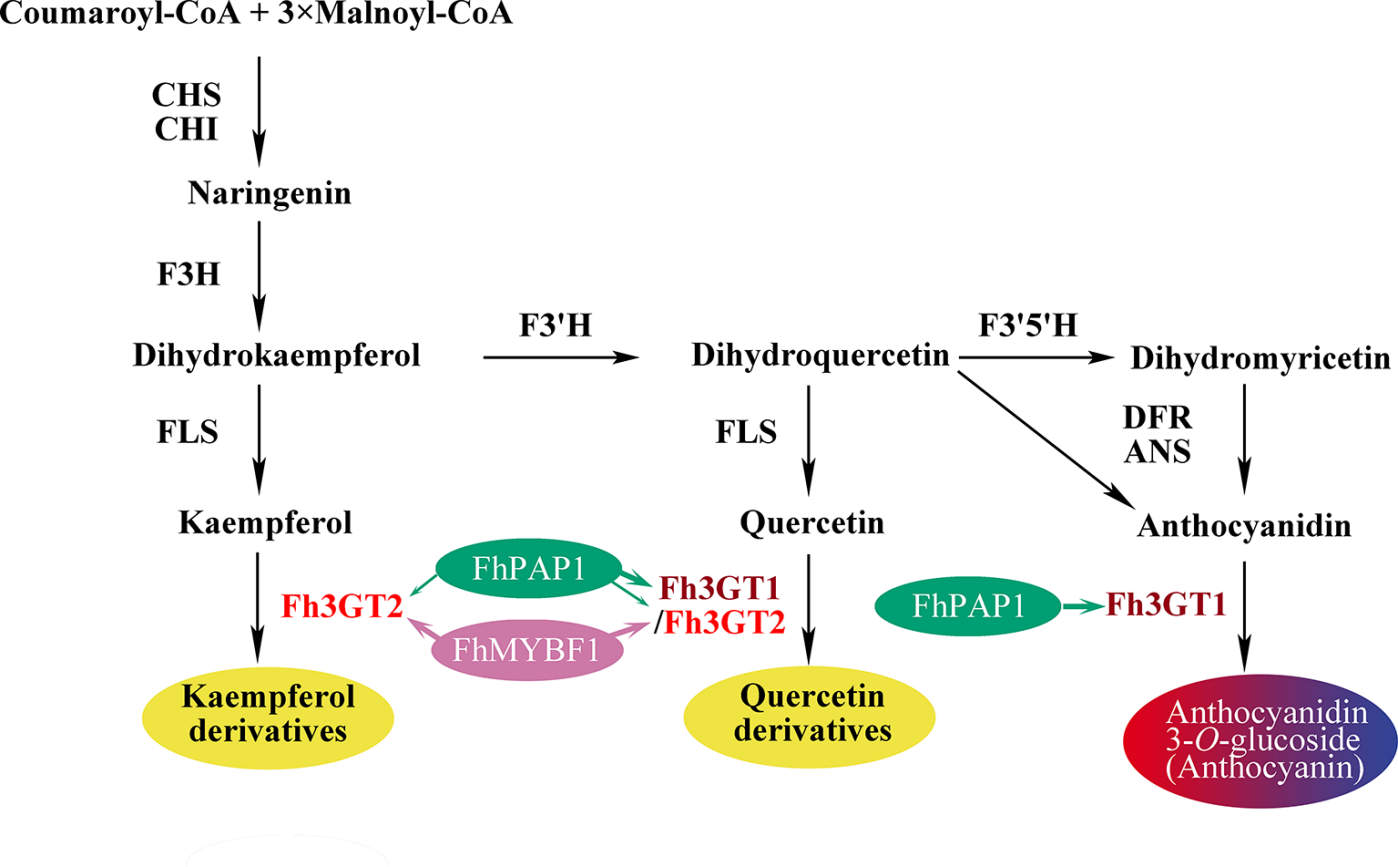
Schematic diagram illustrating the roles of *Fh3GT2* and *Fh3GT1* in the flavonoid biosynthesis of *Fressia hybrida*. Fh3GT2 was mainly responsible for the glycosylation of kaempferol and quercetin, with different efficiencies, while Fh3GT1 preferred to glycosylate anthocyanidins and quercetin. Both Fh3GT2 and Fh3GT1 could be regulated by FhPAP1, with different efficiencies, whereas FhMYBF1 could only activate the expression of *Fh3GT2*. *CHS* chalcone synthase, *CHI* chalcone isomerase, *F3H* flavanone 3-hydroxylase, *F3’, 5’H* ﬂavonoid *3*′, *5'*-hydroxylase, *FLS* flavonol synthase, *DFR* dihydroflavonol reductase, *ANS* anthocyanidin synthase.

Though both Fh3GT1 and Fh3GT2 could glucosylate a series of substrates and showed relatively high activities towards quercetin in sufficient substrates (**Table 1**), they perhaps function divergently. Considering their differential expression patterns, Fh3GT1 and Fh3GT2 might sturdily prefer anthocyanidins and flavonols as substrates, respectively (**Figures 3**, **4**). As glycosyltransferases participated in flavonoid biosynthesis, their *K*_m_ values usually ranged from 0.9 to 400 µM (Owens and Mcintosh, 2009; Veljanovski and Constabel, 2013; Tiwari et al., 2016). The kinetic parameters of the glycosyltransferase assays revealed that the *K*_m_ of Fh3GT2 for kaempferol was even lower than 0.9 µM, suggesting that kaempferol was feasibly a natural substrate for Fh3GT2 *in vivo* (**Table 1**). Further kinetic parameter assays confirmed the existence of a divergence that Fh3GT2 displayed maximum specificity towards kaempferol, while Fh3GT1 efficiently glycosylated quercetin (**Table 2**). As flavonols evolutionarily appeared earlier than anthocyanins, we arbitrarily inferred that the original function of Fh3GT1 might be quercetin glucosylation. The Fh3GT1 neofunctionalization in the glycosylation of anthocyanidins might be a latter scenario during evolution (Ohno, 1971). In many plant species, the flavonol and anthocyanin pathways were usually regulated by different MYB factors, as exemplified in *Arabidopsis* (Baudry et al., 2004; Stracke et al., 2007; Gonzalez et al., 2008; Jaakola, 2013; Pireyre and Burow, 2015; Xu et al., 2015). In another study, two MYB regulators, FhPAP1 and FhMYBF1, involved in anthocyanin and flavonol biosynthesis were functionally characterized (data not published). Transient transfection assays in the present study depicted that Fh3GT1 was activated by FhPAP1 whereas Fh3GT2 was regulated by either FhPAP1 or FhMYBF1, implying the function divergence of Fh3GT1 and Fh3GT2 in transcriptional levels. Previously, the anthocyanin metabolic pathway in *F. hybrida* has been proposed (Sun et al., 2015). Presently, the functional differentiation and regulatory mechanism of *Fh3GT1* and *Fh3GT2* in *F. hybrida* were further perfected (**Figure 7**). Comparatively, *Fh3GT1* primarily activated by FhPAP1 catalyzed the biosynthesis of anthocyanidins and quercetin glycosylation despite the fact that *Fh3GT2* primarily activated by FhMYBF1 or FhPAP1 was central in flavonol glycosylation, especially for kaempferol derivatives. However, steady effort towards deciphering all the possible functions and regulatory elements of the glycosyltransferase gene family in *Freesia* is crucial. For instance, the regiospecificity of Fh3GT2 and whether Fh3GT2 could transfer UDP-galactose or other sugar donors to flavonoid were beyond the scope of the current investigation. Considering the high similarities among Fh3GT3, Fh3GT4, and Fh3GT2, their invalid functions in transferring UDP-glucose to anthocyanidins or flavonols promote further research to decode the mechanisms of Fh3GT3 and Fh3GT4 in glycosylating other flavonoids or metabolites (Fukuchi-Mizutani et al., 2003; Sawada et al., 2005; Yonekura-Sakakibara et al., 2007; Yonekura-Sakakibara et al., 2012; Bönisch et al., 2014b; Dong et al., 2014; Liu et al., 2015; Pasquet et al., 2016; Mageroy et al., 2017). It is conceivable that the present study provided new insights into plant flavonoid modifications, as well as the evolutionary divergence in *UF3GT* paralogous genes in the biochemical and transcriptional levels. Moreover, the plant UFGT promiscuity makes them pretty candidates especially in the exploration of biocatalysts with capability to form region-specific isomers of a specified glycoside for biotechnological use.

## Data Availability Statement

The datasets generated for this study can be found in the Genebank : MK945761.

## Author Contributions

XM, YL, TZ, WS, and XS performed the experiments and helped in analyzing data. YL and XG wrote and revised this manuscript. XG and LW designed the experiments. All authors have participated in this research and approved the final manuscript.

## Funding

This work was supported by the National Natural Science Foundation of China (31900252, 31972445); the China Postdoctoral Science Foundation funded project (2018M641761); the Department of Science and Technology of Jilin Province (20190201299JC, 20190303095SF, 20130604037TC); the Programme for Introducing Talents to Universities (B07017); and the Fundamental Research Fund for the Central Universities. The funders had no role in the study design, data collection and analysis, decision to publish, or preparation of the manuscript.

## Conflict of Interest

The authors declare that the research was conducted in the absence of any commercial or financial relationships that could be construed as a potential conflict of interest.
