# Supplementary material for: Functional Differentiation of Duplicated Flavonoid 3-O-Glycosyltransferases in the Flavonol and Anthocyanin Biosynthesis of Freesia hybrida
Source: Front Plant Sci. 2019 Oct 18;10:1330. doi: 10.3389/fpls.2019.01330 (PMC6813240; doi:10.3389/fpls.2019.01330)
Supplement: Supplementary file 1 [file DataSheet_1.doc]

***Table S1.*** *Information of the 3GT-like genes in Freesia hybrida*

| **Gene** | **ORF (bp)** | **Number of amino acids** | **Molecular weight (kDa)** | **Theoretical pI** | **Function prediction** | **Similarity to *Fh3GT1*** |
| --- | --- | --- | --- | --- | --- | --- |
| *Fh3GT1* | 1338 | 445 | 48 | 5.88 | UDP-  glycosyltransferase | 100% |
| *Fh3GT2* | 1362 | 453 | 48.5 | 7.14 | UDP-  glycosyltransferase | 45.75% |
| *Fh3GT3* | 1371 | 456 | 49.4 | 5.75 | UDP-  glycosyltransferase | 46.01% |
| *Fh3GT4* | 1368 | 455 | 48.6 | 5.33 | UDP-  glycosyltransferase | 43.15% |

**Table S2.** The primers used in present study

|  | Primer name | The prime sequences (5’-3’) |
| --- | --- | --- |
| For ORF PCR | Fh3GT2-ORF F | ATGGGCTCCAGCCAACCT |
|  | Fh3GT2-ORF R | TTAGCCGTTAATAATCTCGAT |
|  | Fh3GT3-ORF F | ATGTCGTCAACAATGGAAC |
|  | Fh3GT3-ORF R | TTAGGCTTTTACAATCTCCAG |
|  | Fh3GT4-ORF F | ATGAGCAGAGGCAGCCATAG |
|  | Fh3GT4-ORF R | CTACACATTAGCATTTCCGC |
| For qRT-PCR | qFh3GT2 F | CTTCACCATCCTATCTCCGTTG |
|  | qFh3GT2 R | CGAGCGTACCGAAACTAACA |
|  | qFh3GT1 F | TCCTCTGGTTCGTTGGGGACATC |
|  | qFh3GT1 R | GTTCCTCAGCATGTCGGTGTAGAG |
|  | qFh3GT3 F | CTCGGCATTGATACCTCATCTC |
|  | qFh3GT3 R | GCTAGGGTCATCATCGTCATTT |
|  | qFh3GT4 F | GATCGGGAAGCTAAGGGATAAG |
|  | qFh3GT4 R | CTCCAGGAGGCTCTTAAAGTTC |
| For protein expression | Fh3GT2-Pr F | TGGCTGATATCGGATCCATGGGCTCCAGCCAACCT |
|  | Fh3GT2-Pr R | CGACGGAGCTCGAATTCTTAGCCGTTAATAATCTCGAT |
|  | Fh3GT3-Pr F | TGGCTGATATCGGATCCATGTCGTCAACAATGGAAC |
|  | Fh3GT3-Pr R | CGACGGAGCTCGAATTCTTAGGCTTTTACAATCTCCAG |
|  | Fh3GT4-Pr F | TGGCTGATATCGGATCCATGAGCAGAGGCAGCCATAG |
|  | Fh3GT4-Pr R | CGACGGAGCTCGAATTCCTACACATTAGCATTTCCGC |
| Protoplast assays | FhMYBF1 F | CTGATTACGCTCATATGATGGTAAGGGCTCCTTGCT |
|  | FhMYBF1 R | AGGATTCAATCTTAAGTCAGAAGATATCTGAAACTAGCCAA |
|  | FhPAP1 F | CTGATTACGCTCATATGATGAAACATCAGTAC |
|  | FhPAP1 R | AGGATTCAATCTTAAGTTAATTTTCAGTTCTC |
|  | ProFh3GT1 F | CCAAGCTTGCATGCCTGCAGTTGATCAGATTCTCATTATT |
|  | ProFh3GT1 R | GCTAAGCTTACCATGAGCTCGGAGCGATCGGCCGATCCCAT |
|  | ProFh3GT1(T1) F | CCAAGCTTGCATGCCTGCAGGGTCTGTATGGTGAATC |
|  | ProFh3GT1(T2 )F | CCAAGCTTGCATGCCTGCAGGAAATCACCAAACAGAACC |
|  | ProFh3GT1(T3) F | CCAAGCTTGCATGCCTGCAGGGCAGCAATTGGCACGTT |
|  | ProFh3GT1(T4) F | CCAAGCTTGCATGCCTGCAGGGCTATTAACTTGTATGTAA |
|  | ProFh3GT2 F | CCAAGCTTGCATGCCTGCAGGGATCACTCTTGATGCCGAGAG |
|  | ProFh3GT2 R | GCTAAGCTTACCATGAGCTCTTGGCTGGAGCCCATTGCAGTAT |
|  | ProFh3GT2(T1) F | CCAAGCTTGCATGCCTGCAGACTAATCTCTTTTGAGAAG |
|  | ProFh3GT2(T2) F | CCAAGCTTGCATGCCTGCAGTAATATATGTGGCAACTCA |
|  | ProFh3GT2(T3) F | CCAAGCTTGCATGCCTGCAGACTGGATATACAATTACTCC |
|  | ProFh3GT2(T4) F | CCAAGCTTGCATGCCTGCAGGTAACCGTTTGCCAAAGG |





**Figure S1.** HPLC and MS analysis of flavonols in *Freesia* cultivar Ambiance.

The main peaks were analyzed by mass spectra and indicated as kaempferol derivatives (mass-to-charge ratio =287) and quercetin derivatives (mass-to-charge ratio =303), respectively.


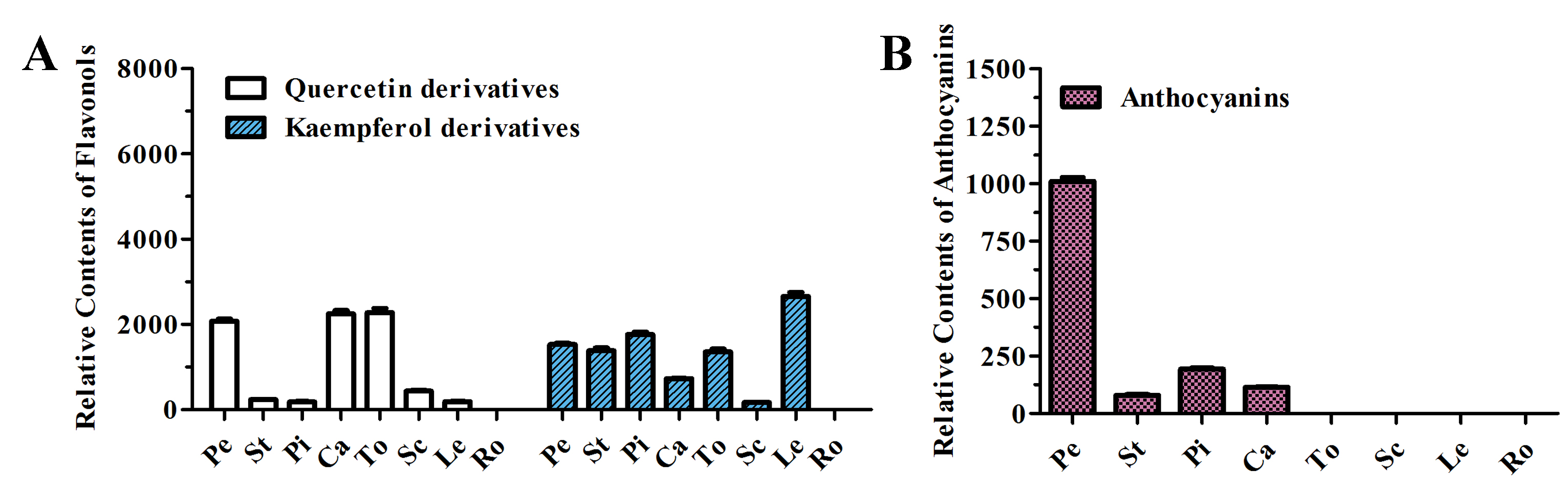


**Figure S2.** Quantitative analysis of anthocyanins and flavonols in different tissues or organs of Red River®.

(A) The relative contents of quercetin derivatives and kaempferol derivatives in different tissues or organs of Red River®. (B) The relative contents of total anthocyanins in different tissues or organs of Red River®. The relative content was represented by the peak area of HPLC analysis. Pe, petals; St, stamens; Pi, pistils; Ca, calyxes; To, toruses; Sc, scapes; Le, leaves; Ro, roots. Data represented means ± SD of three biological replicates.


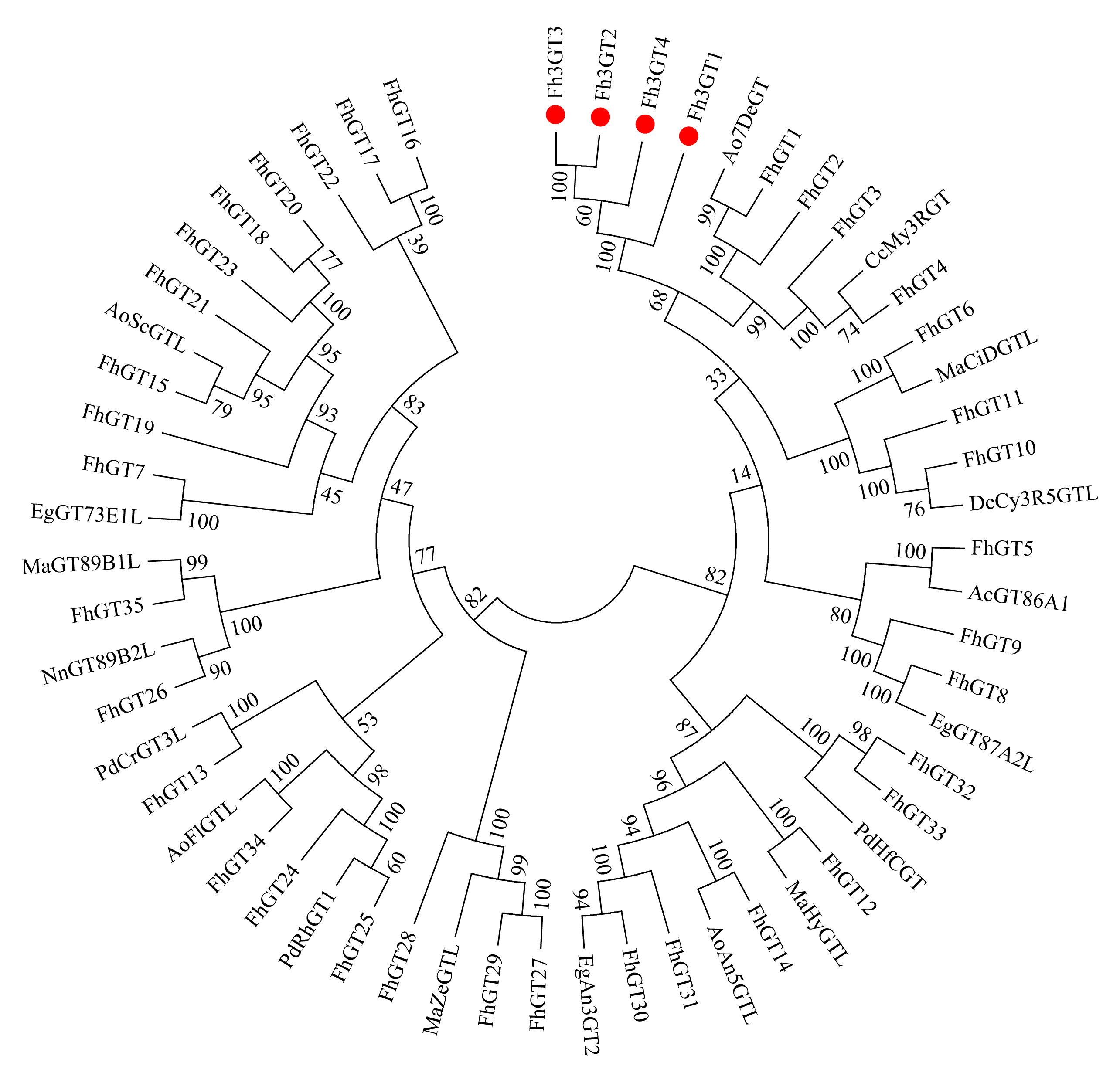


**Figure S3. The phylogenic analysis of the potential glycosyltransferases from *Freesia* and other plants.**

Numbers indicated bootstrap values for 1000 replicates. *Freesia* proteins were tentatively named as FhGTs. The candidate Fh3GTs were indicated by red circles. The GenBank accession numbers of the protein sequences used were as follows: *Freesia hybrida* Fh3GT1 (ADK75021.1), Fh3GT2 (MK945761); *Asparagus officinalis* Ao7DeGT (XP_020255755.1), AoAn5GTL (XP_020249553.1), AoScGTL (XP_020251591.1), AoFlGTL (XP_020263780.1); *Crocosmia x crocosmiiflora* CcMy3RGT (AXB26716.1); *Ananas comosus* AcGT86A1 (XP_020095244.1); *Musa acuminata subsp. malaccensis* MaCiDGTL (XP_009385207.1), MaHyGTL (XP_009419775.1), MaZeGTL (XP_009400942.1), MaGT89B1L (XP_009403270.1); *Elaeis guineensis* EgGT73E1L (XP_010914945.2), EgGT87A2L (XP_010922029.1), EgAn3GT2 (XP_010929083.1); *Dendrobium catenatum* DcCy3R5GTL (XP_020680853.1); *Phoenix dactylifera* PdCrGT3L (XP_008808465.1), PdHfCGT (XP_008791997.1), PdRhGT1 (XP_008788932.1); *Nelumbo nucifera* NnGT89B2L (XP_010261336.1).





**Figure S4. *In vitro* enzyme activity assays of Fh3GT1 towards myricetin in the presence of UDP-glucose.**

The recombinant Fh3GT1 extracted from *E. coli* was reacted with UDP-glucose and the myricetin aglycone. The identification of the products was confirmed based on the standard substance and the relative retention time.


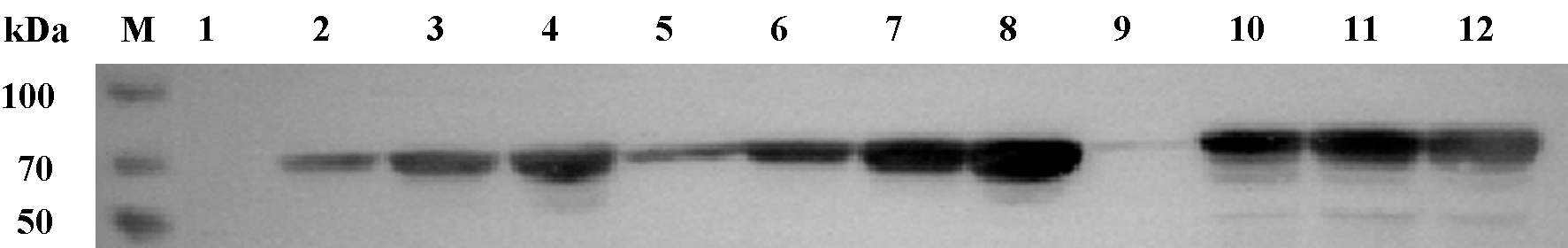


**Figure S5. The analysis of recombinant Fh3GT2, Fh3GT3 and Fh3GT4 proteins by western blotting.**

Lane M, Protein marker; Lane 1-4 represented the proteins extracted from different groups of *E. coli* transformants expressing Fh3GT4, such as the group without IPTG (Lane 1), the group induced by IPTG for 4 h at 37 ℃ (Lane 2), 25 ℃ (Lane 3) and 16 ℃ (Lane 4), respectively. Lane 5-8 and Lane 9-12 represented Fh3GT2 and Fh3GT3 extracted from *E. coli* transformants with similar treatment aforementioned.


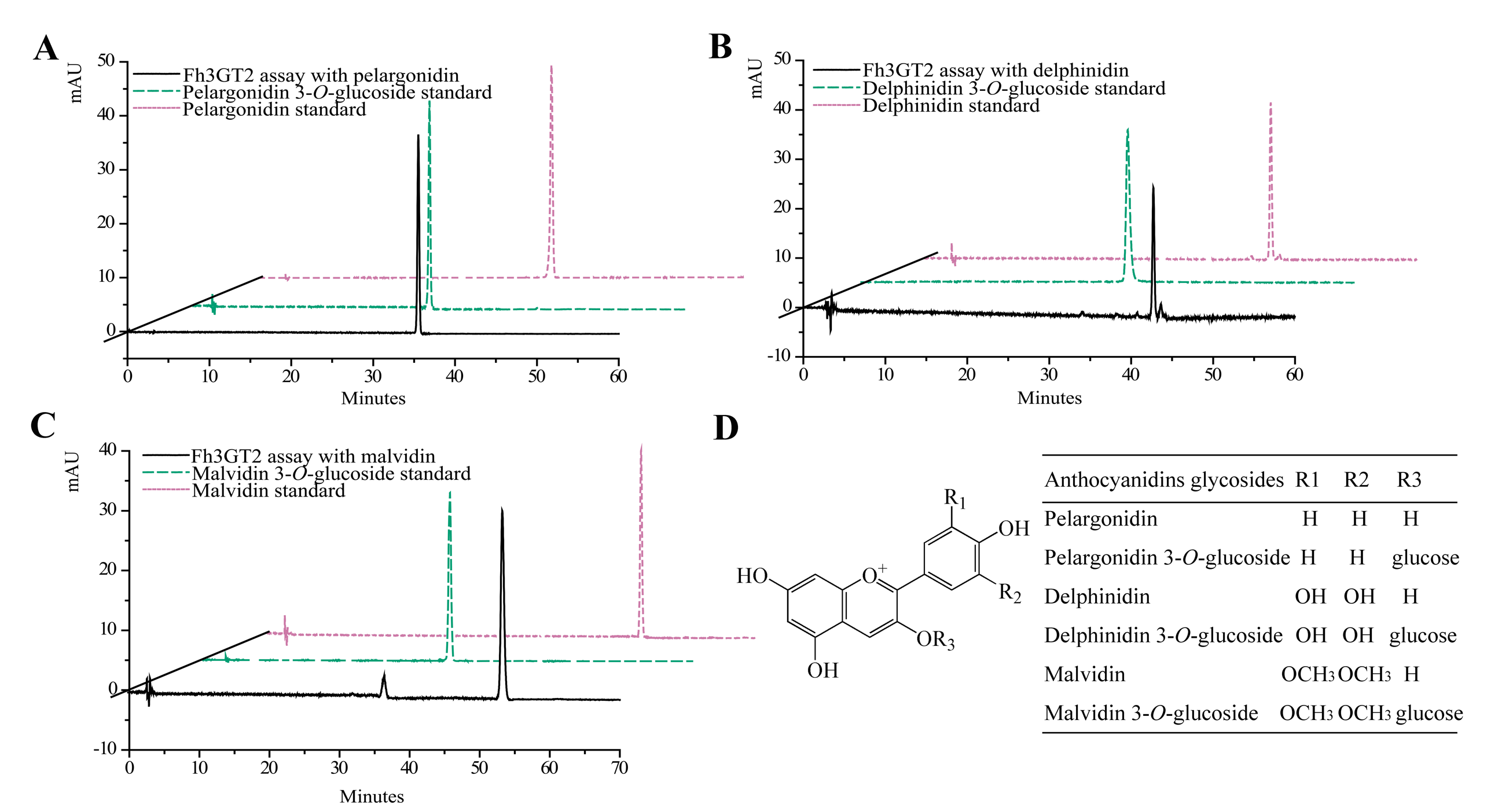


**Figure S6. *In vitro* enzyme activity assays of Fh3GT2 towards pelargonidin, delphinidin and malvidin in the presence of UDP-glucose.**

The recombinant Fh3GT2 extracted from *E. coli* was reacted with UDP-glucose and the respective anthocyanidin aglycone. The identification of the products was confirmed based on the standard substance and the relative retention time.


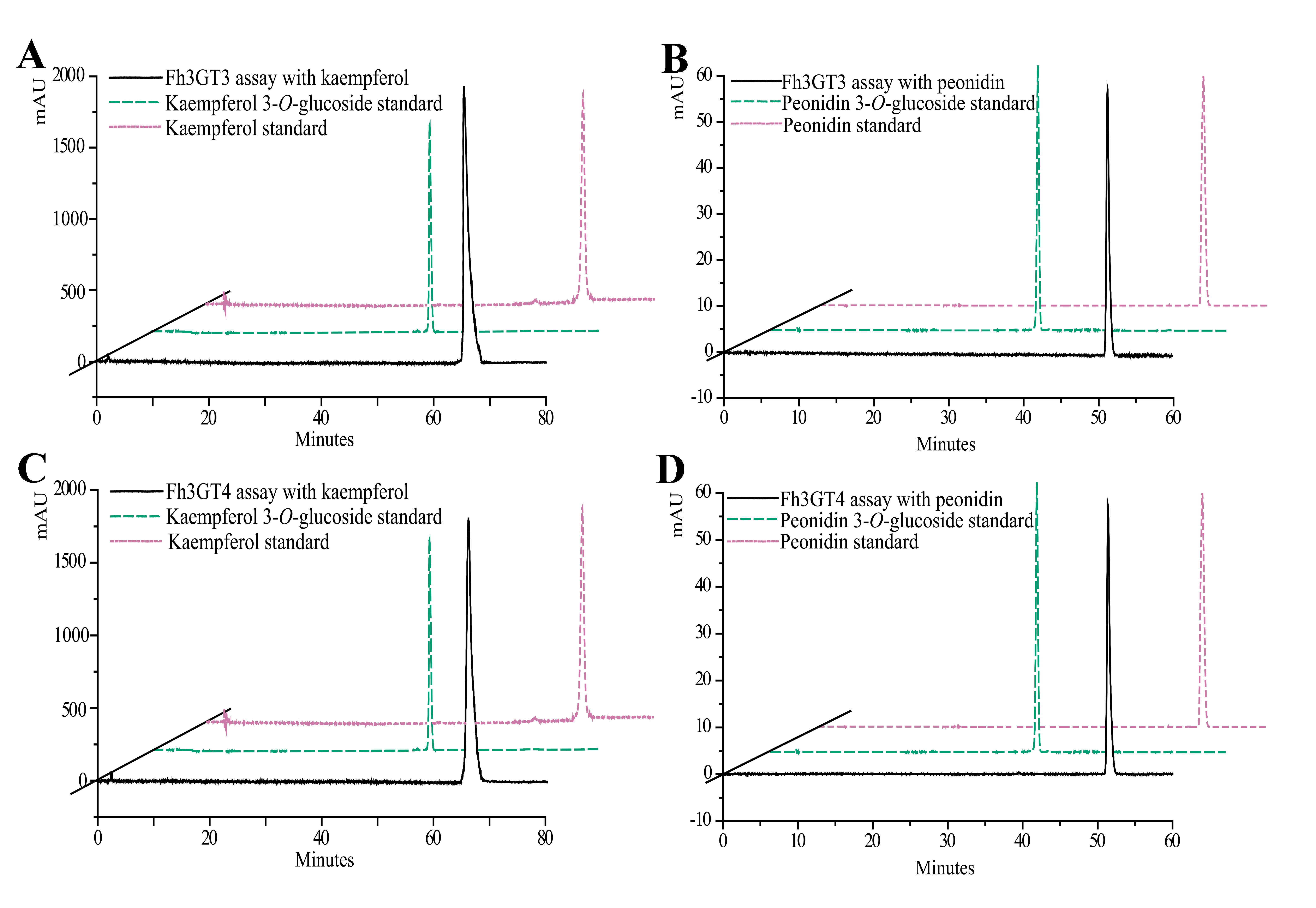


**Figure S7. *In vitro* enzyme activity assays of Fh3GT3 and Fh3GT4 in the presence of UDP-glucose.**

The recombinant Fh3GT3 or Fh3GT4 extracted from *E. coli* was reacted with UDP-glucose and flavonol or anthocyanidin aglycones. As no obvious product was observed in any reactions, only enzyme activities toward kaempferol and peonidin were showed as examples. The identification of the products was confirmed based on the standard substance and the relative retention time.


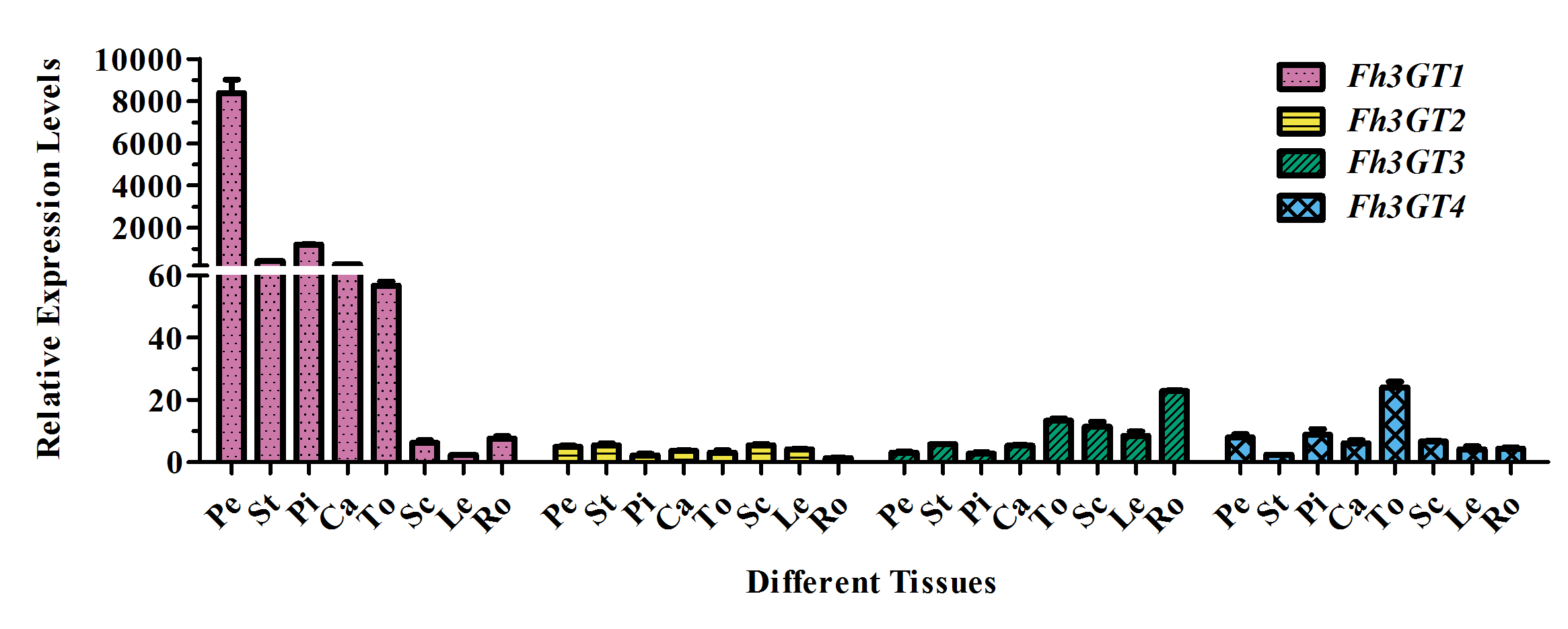


**Figure S8. Expression patterns of *Fh3GT* genes in different tissues or organs of Red River®.**

Pe, petals; St, stamens; Pi, pistils; Ca, calyxes; To, toruses; Sc, scapes; Le, leaves; Ro, roots. Data represented the mean±SD of three replicates.

The promoter sequence of *Fh3GT*

Highlight is the initiation codon

>*ProFh3GT1*

TTGATCAGATTCTCATTATTGAACTTTTGTTTGTCATGACAATGTTAAAATCTCATTGGCCAAATCATAGAGACAGATAAAACAATTTTGATGCCCTCTCCACATTTTTCACCCCTGTTTGCCATTTGATTTGCCATCCAGTTCTGTTTGCCATTTGATTTGTCATCCAACTCCTCCCAAGCAGCTCCTCCCAACATCCAGCACCTCCCAACCAGTAACTGTAAAAGTTTAGTCGAATTGACGATGGAGATGAATGAAAGTATTTCTCGTAGCAAGTTTTTCATATGGAATATACACATAAACTAAAAATGAAAAACAATAGAAATGTCAAAAAAAAAAAAAAATACAGTCATAGTTGTTATATATGAAATGTTATATTTTTTTAATAATAATAATATATTTTGAGATATATATTAAATATAAAGATGGTTTTTGGGATTGCTTTTCCAATCGGTTCTCACTGGAGGAGGTTTTCACCGGAGGGAGGGAACAACTAGTTTGTTTCGTTCTTATACTTTAAAATTCAAATGATCAATTACTCTTTCTTTTAGGGATTCTTTTCGGGAAGAGACAATAGCCTTTTAAAAAAAAAGAAAATAAACACCAATCAGATTATTCCTAAATCAATCTTTTGGTTTGCTTTAAAATTCTTTTCTTTTTTACATTTAAGATTTTGAAAATTCTGGTTGTGGGTTTTGGGTTGGAATGATCAGAAAAAAATGATTATCCTAAAGGTCTGTATGGTGAATCTATAAGGGATCCAAAAGTGTTTTTAACAAGCTAAAAACACTTCCAGTTAAAATATTAAAATATCACTGTTTGGCAAAAGTGTTCATAGATAACTGGACTTTTGAAAATTGAATGCTTTTCTTCACAATTTTGAAATGTCAGTTTTTTTTTTTTTTTTTTGCTTTTCCAGAAAGTACTTTTATAACGTGCTTTTGGATAGAAGCCCATTTGCAATGAAATCACCAAACAGAACCTAAATATAGCATCCAATTAGAAACGCCTGATCTAACACTAATTTTTAACTTTAATTTTATCTCGTATTGCATTTTGATTTATTTAGGGACCATTTATTTACTAACGTCTCTTCTCATCTTGAATATTTCTACAACAATCGTCATTTTCTCTCTATAAACATTTAATTCTATTGCAGTAAGTAAGTGTGAGTCTTCTAATAGATCACGTTGCAACCCCCATTCTCCTGTGGCAGCAATTGGCACGTTGCAATCCCCATTCTCCCTACAAAAACGACGGCAACCACCATCACTATTGATAGTAATTGCATTCTACTTCTAATAGTTATTCTAATAGGCCATGACCGTAAGTCTAGATTATTGATTGACACAATTTGCATTTTACTTCTAATTGGCTATTAACTTGTATGTAACATATAATCAGTTAAGACATCGACACGTTGCAACCCCCATTCTATAGGCACCAACCGATTTTCTGTGTCATTTGTCCCCGCCAACTCTCTTTTAAATACTTCCTCCCCAATTCTTCCAATCACACTCACCACTGCGTCATATCATACTCTTGTTTCTTGCTTGTATTTGATTGATCATCAGCAACAGCAAGCAATGGGATCGGCCGATCGCTCC

>*ProFh3GT2*

GGATCACTCTTGATGCCGAGAGTCATGGAATCGCAATTTAATGTGTAATTCATTTGTTTTGCCAGTTCTCACTGGGTAAATTGGTTCTTTGGCATTTCCAATGCTGCTTTCAATTTGTTGAAATGGCCTTGTGCCTTGTCATTTTCACTTTTTGCCGTTCTTTATTGTTTTTGTGGTAGTGTTGTCATGAGATGCTCATGTTTCTAGTAGCATGATGCTGCAAGGCTCTTTGAGCACATGTAATATGAATGTATGGATTGCAGATTACAAGTACAGTTTGGACTGCATAATTATAAAAATAGAAATCATCTGAGTTTCTGAATTTGTGTTCTGTTGACATTCTTTGTGTCAATTTTATGTATTCTCTATGCAAACAGCATGTGCATATGTGAAACACTAATCTCTTTTGAGAAGATGTTTGAATAAAAAGACGGACGCAAACTGAACCCTCATCATTTTGGTTCCTGCTATTGCAAGATGGTCAATAGTGGATTGGAATGCTATCTGATGATCAAATAGGGAAGATGCCATCGAAAATAGTATAGCTATGTGTGTATTTTTAGCAGATGACATCTAACCCGTTGCTTTACATATTTTCTCTACTATTCTCGAACTGTGGAATGTGCTAGTTATTAGATAAATAATGCATAATATATGTGGCAACTCAAAAGGTAATGATTTGTTTTAACCAAATCCGAGTGAATTTCACGTATCGAAATCCTAGTGGATGATAGCGATATTGATATGATGTCCTTATCGGTCATCAATGATTGAAAACACATGCTACAATAAATAATTTTTTTCAATATACATAGAAGACTTCAAATATATATGAGGGGCTAAATATACATAGCATAATTCCAATATACATAACAGCTCATTTTTCTACTCGAACTCTGCAAAAACACGTTTTCTTCTAACGCCTCGACTCATAACAATTTCTCATGGGCCGAAGGCATTTAAAACTGGATATACAATTACTCCTTTGCCACCTAAAACAATTTTTTTCATTAGTCTAAATACATTAAAACTTAATACCAGGTGGTTATTTGCAATTTGGAAAACATTGGCTTAGTGGTTGGAAGTACAAACTAAAGTAGCTGGCTGGCTGCAATTTTTTTCTAAAACCTAATATATACCTTCCAACTCTTTATAGTTGATTATTATTTTTTATTATAGTCATATCTTCATATATTTTTTAAGTTTCACTTTCTTGTGATCAAGCAAACAAAAGATTAATCACCAAAAACATAGTTTCTTTCATTTTTAGAAACGAAAAAAAGAAAAAAAAAAAAAAGTAACCGTTTGCCAAAGGGGACCAGGTGAACCGTGAACGTGAACTCATACGATGATAGTGGGTACTATCTTTTCGTAACTCTCACCCGTGCGAAGAGAAAGAGACCACGTCGTGCTCCTGATAATGGTAACTACCTTCTCGTGAAGGACTCAAGGACCGAAACTGATTCCGGAGGAATGGAAGATGCGTTACTTACCACGTACTTGAACACCTTCTAGAACACCCCCCACCCCAACGAAATCTGCACCGTCGATTGCCAACAAACTCTTTGGAATCTTTCCTTTAAAAGATATCAAAAGATATCGCAAAGGCCCGAGCTTTCCTCCAACTTCCTTCTTCACTATACTGCAATGGGCTCCAGCCAA
